# Systematic Evaluation of the Viable Microbiome in the Human Oral and Gut Samples with Spike-in Gram+/– Bacteria

**DOI:** 10.1128/msystems.00738-22

**Published:** 2023-03-27

**Authors:** Feng Liu, Hui Lu, Biao Dong, Xiaochang Huang, Hongyu Cheng, Ru Qu, Yichen Hu, Luyun Zhong, Zhenni Guo, Yuehua You, Zhenjiang Zech Xu

**Affiliations:** a State Key Laboratory of Food Science and Technology, Nanchang University, Nanchang, People’s Republic of China; b Jiangxi Agricultural University, College of Bioscience and Bioengineering, Nanchang, People’s Republic of China; c Department of Stomatology, Second Affiliated Hospital of Nanchang University, Nanchang, People’s Republic of China; d Shenzhen Wanhe Pharmaceutical Co., Ltd., Shenzhen, People’s Republic of China; e Department of Stomatology, Longhua People’s Hospital Affiliated to Southern Medical University, Shenzhen, People’s Republic of China; f School of Stomatology, Southern Medical University, Guangzhou, People’s Republic of China; University of Delhi

**Keywords:** PMAxx, live/dead bacteria, host depletion, saliva, feces, freezing, metagenomic sequencing

## Abstract

PMA (propidium monoazide) is one of the few methods that are compatible with metagenomic sequencing to characterize the live/intact microbiota. However, its efficiency in complex communities such as saliva and feces is still controversial. An effective method for depleting host and dead bacterial DNA in human microbiome samples is lacking. Here, we systematically evaluate the efficiency of osmotic lysis and PMAxx treatment (lyPMAxx) in characterizing the viable microbiome with four live/dead Gram+/Gram– microbial strains in simple synthetic and spiked-in complex communities. We show that lyPMAxx-quantitative PCR (qPCR)/sequencing eliminated more than 95% of the host and heat-killed microbial DNA and had a much smaller effect on the live microbes in both simple mock and spiked-in complex communities. The overall microbial load and the alpha diversity of the salivary and fecal microbiome were decreased by lyPMAxx, and the relative abundances of the microbes were changed. The relative abundances of *Actinobacteria*, *Fusobacteria*, and *Firmicutes* in saliva were decreased by lyPMAxx, as was that of *Firmicutes* in feces. We also found that the frequently used sample storage method, freezing with glycerol, killed or injured 65% and 94% of the living microbial cells in saliva and feces, respectively, with the *Proteobacteria* phylum affected most in saliva and the *Bacteroidetes* and *Firmicutes* phyla affected most in feces. By comparing the absolute abundance variation of the shared species among different sample types and individuals, we found that sample habitat and personal differences affected the response of microbial species to lyPMAxx and freezing.

**IMPORTANCE** The functions and phenotypes of microbial communities are largely defined by viable microbes. Through advanced nucleic acid sequencing technologies and downstream bioinformatic analyses, we gained an insight into the high-resolution microbial community composition of human saliva and feces, yet we know very little about whether such community DNA sequences represent viable microbes. PMA-qPCR was used to characterize the viable microbes in previous studies. However, its efficiency in complex communities such as saliva and feces is still controversial. By spiking-in four live/dead Gram+/Gram– bacterial strains, we demonstrate that lyPMAxx can effectively discriminate between live and dead microbes in the simple synthetic community and complex human microbial communities (saliva and feces). In addition, freezing storage was found to kill or injure the microbes in saliva and feces significantly, as measured with lyPMAxx-qPCR/sequencing. This method has a promising prospect in the viable/intact microbiota detection of complex human microbial communities.

## INTRODUCTION

Compared to amplicon sequencing that targets specific genomic regions, metagenomic sequencing provides a comprehensive understanding of the microbiome by cataloging bacterial, fungal, and viral genes. However, this untargeted approach sequences all DNAs within a sample, including host DNA (1). The host genome is usually much larger than the microbial genomes, resulting in high sequencing depth requirements to obtain sufficient microbial reads, especially in samples with high host DNA contamination, such as mucosa, skin, and saliva (2). In addition, all DNAs are sequenced and included in downstream analyses, no matter whether they were from live or dead cells. Dead bacteria were reported to account for 32% of the total bacteria in fresh feces (3). Their DNA can persist for up to a year in the laboratory (4). However, the functions and phenotypes of the microbial community are largely determined by viable microorganisms (1). Relic DNA can obscure treatment effects, spatiotemporal patterns, and relationships between taxa and environmental conditions (5). Thus, removing host DNA and distinguishing live microorganisms from dead is crucial for human metagenomic microbiome studies.

Nelson et al. described a method for reducing human cellular and extracellular DNA in a complex respiratory sample using hypotonic lysis and endonuclease digestion, in which effective microbial sequencing depth was increased and bias introduced into subsequent phylogenetic analysis by bacterial extracellular DNA was reduced (6). Osmotic lysis (selective lysis of mammalian cells) and propidium monoazide (PMA) treatment (lyPMA) were reported to be more effective in removing host-derived sequencing reads than host depletion kits (enzymatic digestion of exposed DNA) and size filtration (7). PMA is a photoreactive DNA-binding dye that preferentially binds to exposed double-stranded DNA (dsDNA). It is one of the few methods that are compatible with metagenomic sequencing to detect live microbes (8) and has been used in discriminating live from dead microbes in infectious diseases (9, 10), food (11–13), and environmental pollution (14, 15). Rogers et al. reported that PMA treatment revealed significant reduction of Pseudomonas aeruginosa load that would otherwise go undetected when measuring the impact of antibiotic therapy on Pseudomonas aeruginosa load in cystic fibrosis respiratory samples (16). PMA sequencing was also used in the detection of the living microbiota in some unique built environments such as the International Space Station (17, 18).

The efficiency of PMA is affected by its concentration, incubation conditions, and sample types (19–21). The efficacy of PMA sequencing in quantifying the live and dead microbes of human microbiome samples was still controversial. Mancabelli et al. showed a 73% reduction in the sequencing reads corresponding to the added free DNA with PMAxx (an improved version of PMA) treatment in saliva, and the percentage of the added free DNA observed in the fecal sample increased with PMAxx treatment (22). Wang et al. evaluated the efficiency of PMA-16S rRNA amplicon sequencing in several complex environmental communities (computer screens, computer mice, soil, and human saliva). They revealed that the efficacy of PMA in removing dead bacteria in saliva samples was relatively low, which might be due to the influence of the large amount of free DNA from human cells (23). Papanicolas et al. reported that PMA treatment efficacy was improved markedly with appropriate sample dilution for fecal samples, which had a high bacterial load and sample turbidity (24). How to effectively deplete host and dead bacterial DNA simultaneously using the PMA method has not been systematically studied in metagenomic sequencing.

Freeze-thaw is an important factor impacting the viability of the microbes during sample collection and storage. A recent study analyzed long-term frozen fecal suspensions with glycerin as a cryoprotectant and found that the abundance of *Bacteroidetes* decreased in a storage duration-dependent manner with DNase pretreatment and sequencing (25). Another study revealed that freeze-thaw reduced microbial viability of FMT (fecal microbiota transplantation) donors’ feces from 50% to 23% using PMA-16S rRNA gene amplicon sequencing (26). As keeping the microbiota alive is important during sample collection and storage, especially in FMT and culture-dependent experiments, exploring how freeze-thaw affected the viability of the microbiota is important.

The mouth and gut are two main ecological niches with high microbial diversity and play important roles in human health (27, 28). We evaluated the effect of lyPMAxx on depleting both host and dead bacterial DNA in human salivary and fecal samples. PMAxx is a new and improved version of PMA which is more effective at eliminating PCR amplification of dead cell DNA and has better results than PMA in distinguishing viable from dead bacteria (29) (https://biotium.com/technology/pma-for-viability-pcr/#pmaxx). Here, we first validated the working conditions of lyPMAxx using a simple synthetic community, which contained Caco2 cells (a human colorectal adenocarcinoma cell line) mixed with four live/dead Gram+/Gram– strains. The efficiency of lyPMAxx was next studied and validated in spiked-in complex human salivary and fecal samples. The response of the endogenous microbiota to lyPMAxx and freeze-thaw was analyzed in different individuals and sample types. Our results showed that lyPMAxx sequencing could effectively eliminate host and dead bacterial DNA and characterize the viable microbes in both spiked-in saliva and feces. The effect of lyPMAxx was repeatable on the replicate samples. Salivary and fecal microbial species respond to lyPMAxx and freeze-thaw treatment depending on individual differences and sample types.

## RESULTS

### LyPMAxx effectively removed exposed DNA in simple mock and complex microbial communities.

**FIG 1:**
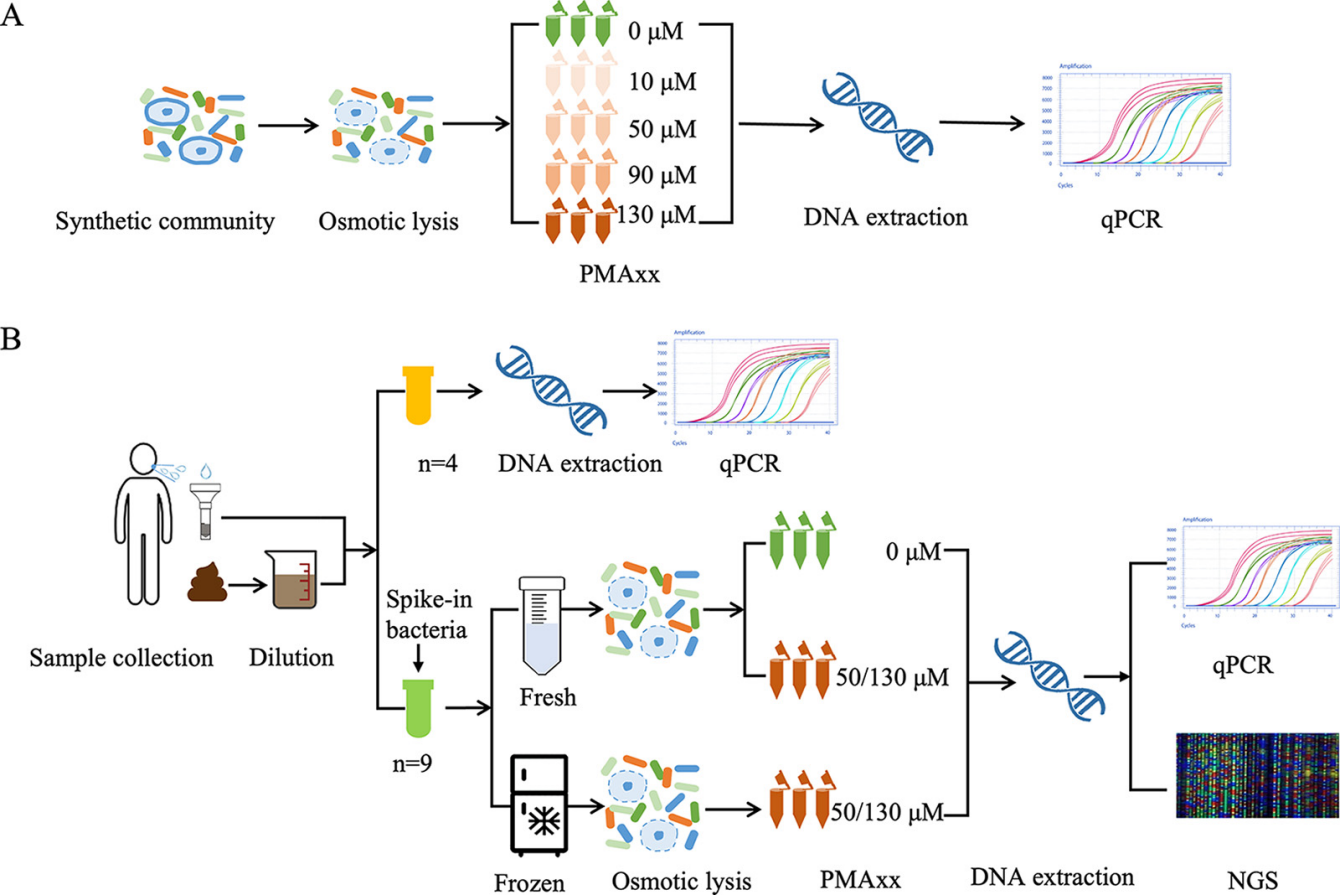
LyPMAxx treatment procedures of the synthetic mock community, saliva, and feces samples. (A) LyPMAxx treatment procedures of the synthetic community. (B) LyPMAxx treatment procedures of the spiked-in saliva and feces samples. For each group from each person, three technical replicates were performed.

**FIG 2:**
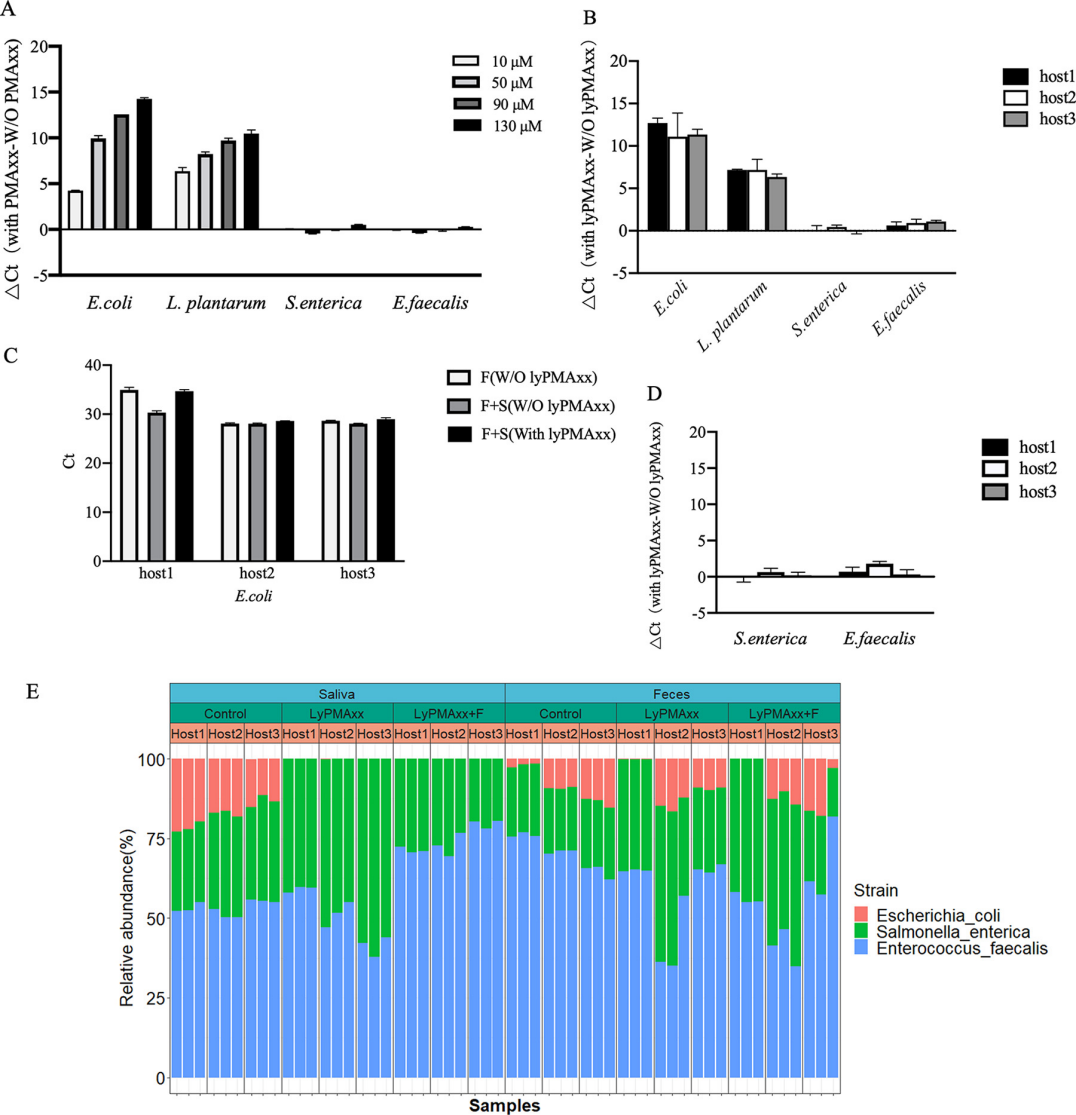
LyPMAxx inhibited the amplification of dead bacterial DNA and had a much smaller influence on live bacteria in the simple synthetic community, spiked-in saliva, and feces samples. (A) Effect of lyPMAxx on the bacteria of the synthetic community. (B) Effect of lyPMAxx on the spiked-in bacteria in saliva samples. (C) Effect of lyPMAxx on the spiked-in *E. coli* in feces samples: feces samples [F(W/O lyPMAxx)], feces samples plus spike-in cultures [F+S(W/O lyPMAxx]), feces samples plus spike-in cultures plus lyPMAxx treatment [F+S(With lyPMAxx)]. (D) Effect of lyPMAxx on the spiked-in *S. enterica* and *E. faecalis* in feces samples. (E) The relative abundance of the spiked-in bacteria in saliva and feces samples by shotgun metagenomic sequencing.

A synthetic community was constructed to evaluate the effect of lyPMAxx on the host and dead bacterial DNA depletion. The simple mock community was constituted of four strains, E. coli (dead, Gram–), Lactiplantibacillus plantarum (dead, Gram+), Salmonella enterica (live, Gram–), and Enterococcus faecalis (live, Gram+), as well as Caco2, representing host cells (Table 1). We first tested the optimal PMAxx concentration (0, 10, 50, 90, and 130 μM) in the mock community (Fig. 1A). The bacterial biomass was estimated using quantitative PCR (qPCR) with the species-specific primers. Treatment with 10 μM PMAxx removed 94.71% of dead E. coli and 98.75% of dead *L. plantarum* (Fig. 2A). When the concentration increased to 50 μM or higher, more than 99% of the dead cells were depleted, indicating a dose-dependent manner of lyPMAxx in removing the dead bacteria. Meanwhile, we noticed that lyPMAxx treatment exhibited little effect on live S. enterica and E. faecalis (Fig. 2A; see Table S1 In the supplemental material). Together, our results showed that 50- to 130-μM concentrations of lyPMAxx effectively removed the dead bacteria while having little influence on the viable bacteria in the simple community.

**TABLE 1:** Composition of the synthetic communities

Cell	Gram classification	Synthetic cellular community (/mL)	Spike-in bacterial community (CFU/mL saliva)	Spike-in bacterial community (CFU/g feces)
Caco2		2.00 × 10^5^	0	0
E. coli (dead)	G^−^	2.00 × 10^8^	1.98 × 10^6^	3.94 × 10^7^
*L. plantarum* (dead)	G^+^	2.00 × 10^8^	1.32 × 10^7^	2.62 × 10^8^
S. faecalis (live)	G^+^	2.00 × 10^8^	9.44 × 10^6^	1.88 × 10^8^
S. enterica (live)	G^−^	2.00 × 10^8^	1.09 × 10^7^	2.18 × 10^8^

10.1128/msystems.00738-22.6 TABLE S1 *C_T_* value of Caco2 and the four bacteria in the synthetic cellular community. Download Table S1, DOCX file, 0.01 MB. Copyright © 2023 Liu et al. 2023 Liu et al. https://creativecommons.org/licenses/by/4.0/ This content is distributed under the terms of the Creative Commons Attribution 4.0 International license.

To further evaluate the performance of lyPMAxx in removing dead bacterial cellular DNA in complex human microbial communities, we spiked a well-defined synthetic bacterial community (Table 1) into saliva and feces samples (Fig. 1B). The abundance of the four spike-in strains in the groups with lyPMAxx and without lyPMAxx treatment were then compared using qPCR and metagenomic sequencing. The four spike-in strains were not found in the native saliva of the three hosts as validated by qPCR (Table S2). The threshold cycle (*C_T_*) value of the spike-in dead E. coli (from 24.30 ± 0.12 to 36.01 ± 1.34) and *L. plantarum* (from 29.13 ± 0.24 to 36.03 ± 0.56) increased after lyPMAxx treatment (Fig. 2B, Table S3), indicating that more than 99% of the dead cells were depleted. With shotgun metagenomic sequencing, the relative abundance of E. coli was found to decrease from 22.43% ± 5.40% to 0 in the spiked-in saliva after lyPMAxx treatment (Fig. 2E). We found that Gram-positive bacterium *L. plantarum* is heavily underestimated, which might be due to the low DNA extraction efficiency (Fig. S1, Text S1), resulting in biased quantification of this bacterium. The low DNA extraction efficiency of this bacterium is in agreement with a previous study (38). The spike-in *L. plantarum* could not be detected in saliva and feces using shotgun metagenomic sequencing. The effect of lyPMAxx on live bacteria was relatively small (*C_T_* value increased by 0.88 ± 0.37 for E. faecalis and 0.38 ± 0.19 for S. enterica) (Fig. 2B, Table S3). The ratio of E. faecalis to S. enterica was 1.77 ± 0.39 and 1.05 ± 0.40, respectively, before and after lyPMAxx treatment in the spiked-in saliva (Fig. 2E). The changes of the relative amounts of the live bacteria were limited compared to the ratio of live to dead, which further indicated the ability of lyPMAxx discriminate between live and dead bacteria.

10.1128/msystems.00738-22.2 FIG S1 DNA extraction efficiency was affected by bacterial species, DNA extraction kits, and sample types. (A) DNA yield from three bacterial specimens using two commercial kits. (B) DNA extraction efficiency from three bacterial specimens using two commercial kits, TaKaRa MiniBEST bacterial genomic DNA extraction kit and Tiangen TIANamp stool DNA kit. (C) Spike-in bacterial DNA extraction efficiency in saliva. (D) Spike-in bacterial DNA extraction efficiency in feces. The values are expressed as the means ± SEM. *, *P* < 0.05; **, *P* < 0.01; ***, *P* < 0.001; ****, *P* < 0.0001. Download FIG S1, TIF file, 2.6 MB. Copyright © 2023 Liu et al. 2023 Liu et al. https://creativecommons.org/licenses/by/4.0/ This content is distributed under the terms of the Creative Commons Attribution 4.0 International license.

10.1128/msystems.00738-22.7 TABLE S2 *C_T_* values of the four bacteria in native saliva and fecal samples. Download Table S2, DOCX file, 0.01 MB. Copyright © 2023 Liu et al. 2023 Liu et al. https://creativecommons.org/licenses/by/4.0/ This content is distributed under the terms of the Creative Commons Attribution 4.0 International license.

10.1128/msystems.00738-22.8 TABLE S3 *C_T_* value of spike-in bacteria in saliva and fecal samples before and after lyPMAxx treatment. Download Table S3, DOCX file, 0.01 MB. Copyright © 2023 Liu et al. 2023 Liu et al. https://creativecommons.org/licenses/by/4.0/ This content is distributed under the terms of the Creative Commons Attribution 4.0 International license.

Similarly, the efficiency of lyPMAxx was also assessed in feces. E. coli was highly abundant in native feces of hosts 2 and 3 but its abundance in host 1 was relatively low (Tables S2 and S4). We thus used the feces of host 1 to determine the efficiency of lyPMAxx in removing dead bacteria. The *C_T_* value of E. coli increased from 30.33 ± 0.36 to 34.70 ± 0.31 after lyPMAxx treatment, indicating that 95.16% of dead E. coli was removed (Fig. 2C and Table S4), and the relative abundance of E. coli decreased from 1.92 ± 0.62% to 0.07 ± 0.02% in feces of host 1 (Fig. 2E). *L. plantarum* could not be detected by qPCR and shotgun metagenomic sequencing in feces, which might be due to low DNA extraction efficiency (Fig. S1 and Table S3). A much smaller effect was shown on the live bacteria (the *C_T_* value increased by 0.97 ± 0.78 for E. faecalis and 0.37 ± 0.50 for S. enterica) in three hosts’ feces, and the ratio of E. faecalis to S. enterica was 3.33 ± 0.31 and 2.05 ± 0.49 before and after lyPMAxx treatment, respectively (Fig. 2E).

10.1128/msystems.00738-22.9 TABLE S4 *C_T_* value of *E. coli* in feces samples before and after lyPMAxx treatment. Download Table S4, DOCX file, 0.01 MB. Copyright © 2023 Liu et al. 2023 Liu et al. https://creativecommons.org/licenses/by/4.0/ This content is distributed under the terms of the Creative Commons Attribution 4.0 International license.

Collectively, we concluded that lyPMAxx could eliminate more than 95% of the spike-in dead bacteria in saliva and feces and had a relatively smaller influence on the live bacteria. The effect of lyPMAxx on spike-in live and dead bacteria was relatively consistent in saliva and feces.

### LyPMAxx treatment significantly eliminated the contamination of host DNA.

**FIG 3:**
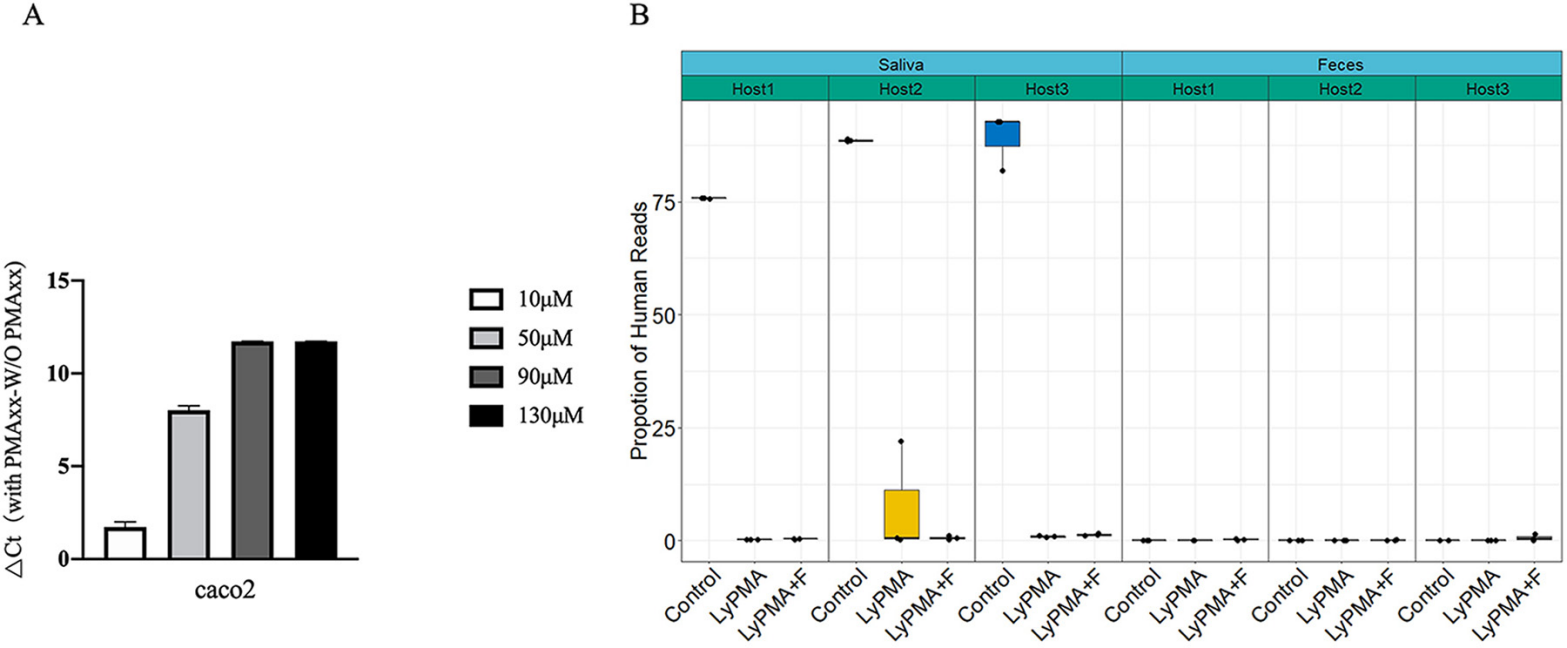
Host DNA depletion by lyPMAxx in synthetic community, saliva, and feces samples. (A) Caco2 DNA depletion by lyPMAxx was measured with qPCR. (B) The variation of host DNA proportion in saliva and feces by lyPMAxx.

We also measured the effect of lyPMAxx on host cells. qPCR showed that 10 μM lyPMAxx removed 70.32% of Caco2 cellular DNA in the simple synthetic community, and 50 μM lyPMAxx increased the removal efficiency to 99.61% (Fig. 3A), indicating that lyPMAxx treatment could reduce the amplification of the Caco2 cellular DNA in a dose-dependent manner.

The proportion of human reads was as high as 84.49 ± 7.55% in saliva, and it decreased to 0.50 ± 0.33% and 0.77 ± 0.49% in fresh and frozen saliva samples, respectively, after lyPMAxx treatment (Fig. 3B), indicating that lyPMAxx treatment could effectively remove the host reads in saliva as previously reported (7). Fecal samples had low levels of host contamination (0.11% ± 0.01). No obvious change in host read proportion was observed after lyPMAxx treatment (Fig. 3B).

### Microbiota alteration by lyPMAxx treatment in fresh saliva and feces samples.

**FIG 4:**
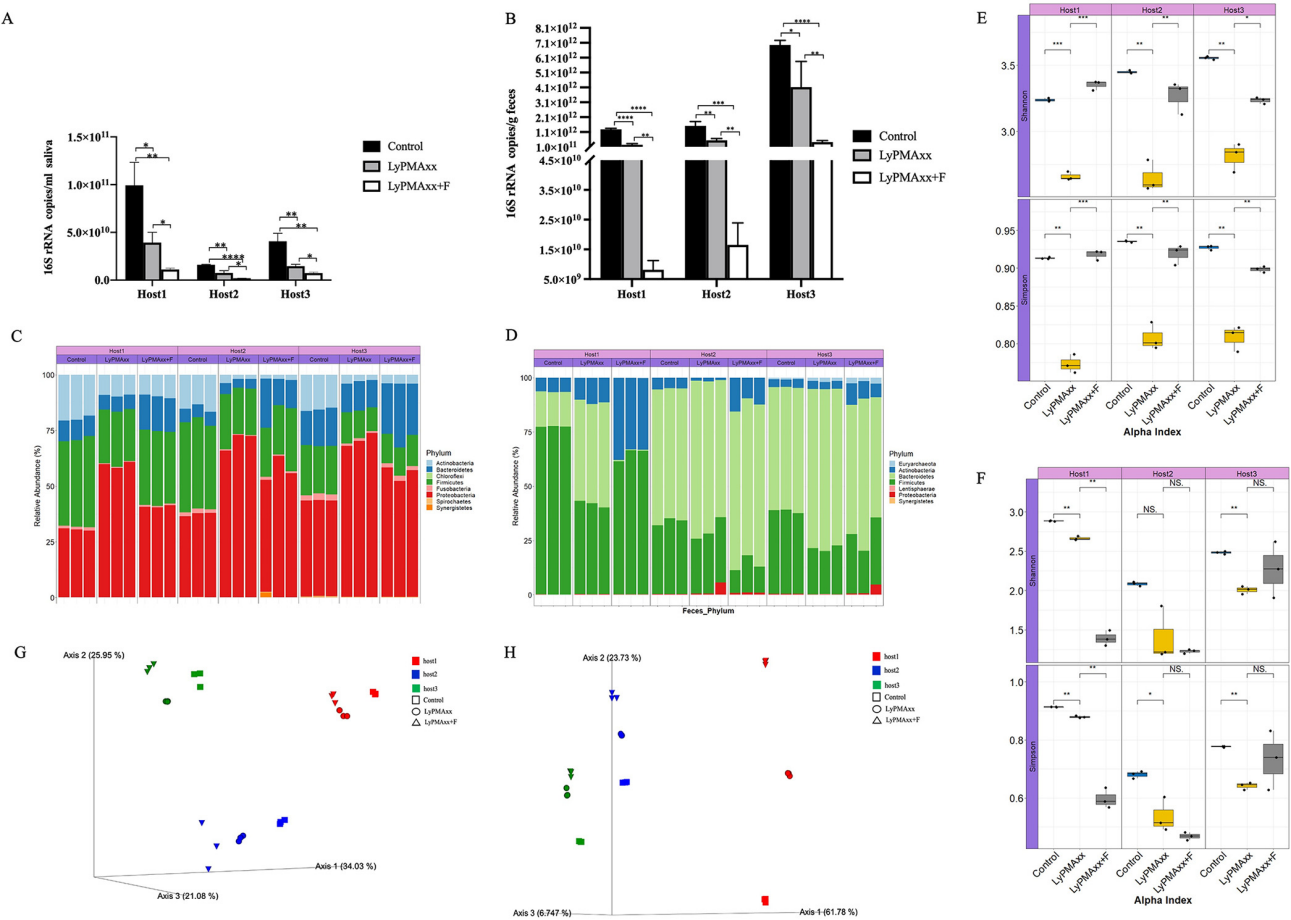
Effect of lyPMAxx and freezing on the microbiota of saliva and feces. (A and B) Total 16S rRNA gene copy numbers were decreased significantly after lyPMAxx and freezing treatment in saliva (A) and feces (B). (C and D) Variation of the microbiota composition at the phylum level in saliva (C) and feces (D). (E and F) Variation of alpha diversity (Shannon and Simpson indexes) is represented as boxplots in saliva (E) and feces (F). (G and H) Clustering of samples by PCoA based on Bray-Curtis similarity in saliva (G) and feces (H). Significant differences were identified by paired *t* tests for 16S rRNA gene copy numbers and the Wilcoxon test for alpha diversity. The values were expressed as the means ± SEM. *, *P* < 0.05; **, *P* < 0.01; ***, *P* < 0.001; ****, *P* < 0.0001.

We next compared the bacterial diversity and composition of control and lyPMAxx treatment groups to declare the effect of lyPMAxx on the salivary and fecal microbiota. First, the microbial loads were quantified. With lyPMAxx treatment, the microbial loads in fresh saliva decreased to 39.8%, 44.7%, and 36.3% of that in the untreated group in three hosts, respectively (Fig. 4A); in fecal samples, it decreased to 18.3%, 36.1%, and 55.0% of the control group in three hosts (Fig. 4B). Averages of 59.7% and 63.5% of the microbes were dead/injured in saliva and feces, respectively. The magnitude of microbial load change was similar among individuals in saliva samples, while it varied in feces. The microbiome composition of the lyPMAxx treatment group changed compared to that in the control group in both saliva (Fig. 4C) and feces (Fig. 4D). The proportion of *Proteobacteria* dramatically increased, while those of *Firmicutes* and *Actinobacteria* decreased in saliva (Fig. 4C). In feces, the relative abundance of *Firmicutes* was reduced, and that of *Bacteroidetes* was increased. We next investigated the changes in alpha diversity between samples with and without lyPMAxx treatment. We found that the Shannon and Simpson indexes were significantly reduced after lyPMAxx treatment in both saliva (Fig. 4E) and feces samples (Fig. 4F). Further, a principal-coordinate analysis (PCoA) plot based on the Bray-Curtis distance showed that samples from the same individual were clustered regardless of the treatments in both saliva (Fig. 4G; *R* = 0.971, *P* = 0.001) and feces (Fig. 4H; *R* = 0.788, *P* = 0.001). Further statistical analysis of the Bray-Curtis distance between groups of intraindividual samples and interindividual samples showed that lyPMAxx affected the microbiota, but the influence was smaller than individual differences across three hosts (Fig. S3). Among three individuals, the effect of lyPMAxx on fecal microbiota structure was similar in feces of hosts 2 and 3 but larger in that of host 1, indicating that the extent to which lyPMAxx affected the microbiota depended on the indigenous microbes in different persons (Fig. S3B).

10.1128/msystems.00738-22.4 FIG S3 Bray-Curtis dissimilarity distributions between intraindividual and interindividual salivary or fecal samples. (A) Bray-Curtis dissimilarity distributions between intraindividual and interindividual salivary or fecal samples. (B) Bray-Curtis dissimilarity distributions between intraindividual and interindividual fecal samples. Download FIG S3, TIF file, 1.9 MB. Copyright © 2023 Liu et al. 2023 Liu et al. https://creativecommons.org/licenses/by/4.0/ This content is distributed under the terms of the Creative Commons Attribution 4.0 International license.

**FIG 5:**
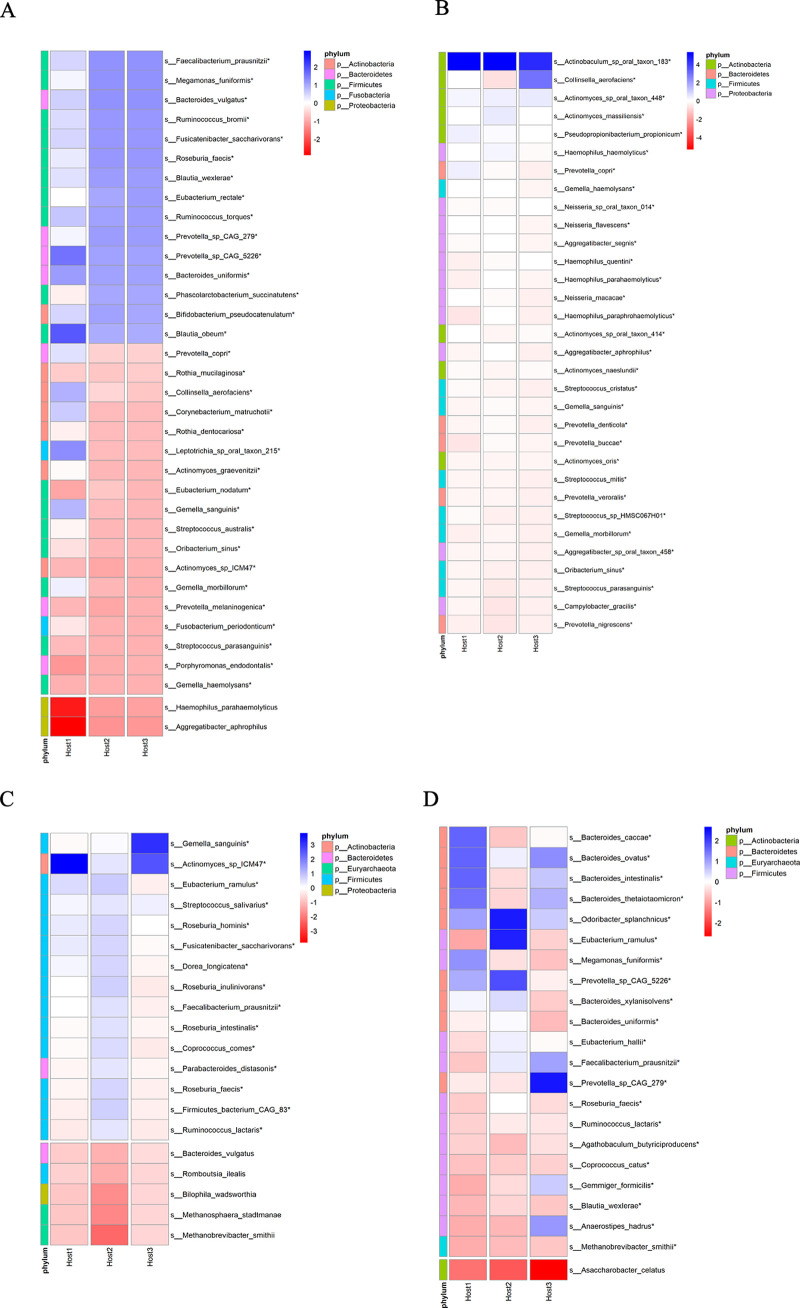
Effect of lyPMAxx and freezing on the shared species in different individuals. (A and C) Fold changes in the absolute abundance of the representative bacterial species in saliva (A) and feces (C) of three hosts are represented as heatmaps. The species with fold changes larger than 4 times (top) were separated from those decreased less than 2.5 times (bottom). (B) Freezing-responsive species with fold changes bigger than 10 times in saliva of the three hosts. (D) Freezing -responsive and freezing -resilient species in feces of the three hosts are shown. The species with fold changes bigger than 25 times (top, freezing -responsive species) or less than 2.5 times (bottom, freezing-resilient species) were included.

To investigate the influence of lyPMAxx on the microbiota in saliva and feces among three individuals, we compared the abundance at the species level between groups. The variation in the relative abundance of the species is shown in Fig. S2. Taking account of the variation in bacterial load among three individuals, the inferred absolute abundance for each bacterium taxon was calculated as mentioned in Materials and Methods. Among the 102 saliva-shared species and the 63 feces-shared species, 70 and 53 of these species, respectively showed significant declines in inferred absolute abundance (Table S5). We found that the majority of the most significantly changed species in abundance belong to the *Firmicutes* phylum (Fig. 5A and C, top panel). The species least affected by lyPMAxx are shown at the bottom of Fig. 5A and C. The effect of lyPMAxx on the bacterial species was dependent on the personal difference. For example, Faecalibacterium prausnitzii and Megamonas funiformis in saliva decreased significantly by lyPMAxx in all three hosts, but they were more sensitive to the treatment in hosts 2 and 3 than that in host 1. Haemophilus parahaemolyticus and Aggregatibacter aphrophilus were more resistant to lyPMAxx in host 1 than in hosts 2 and 3. The live/dead ratio of the shared microbial species in saliva was more similar between hosts 2 and 3 than host 1 (Fig. 5A).

10.1128/msystems.00738-22.3 FIG S2 Variation in the relative abundance of the microbes at the species level by lyPMAxx and freezing in saliva and feces. (A) Variation in the relative abundance of the microbes at the species level by lyPMAxx and freezing in saliva. (B) Variation in the relative abundance of the microbes at the species level by lyPMAxx and freezing in feces. Download FIG S2, TIF file, 1.5 MB. Copyright © 2023 Liu et al. 2023 Liu et al. https://creativecommons.org/licenses/by/4.0/ This content is distributed under the terms of the Creative Commons Attribution 4.0 International license.

10.1128/msystems.00738-22.10 TABLE S5 Fold changes in the absolute abundance of the shared species in the three host saliva and feces samples between saliva and feces by lyPMAxx and freezing. Download Table S5, XLS file, 0.1 MB. Copyright © 2023 Liu et al. 2023 Liu et al. https://creativecommons.org/licenses/by/4.0/ This content is distributed under the terms of the Creative Commons Attribution 4.0 International license.

**FIG 6:**
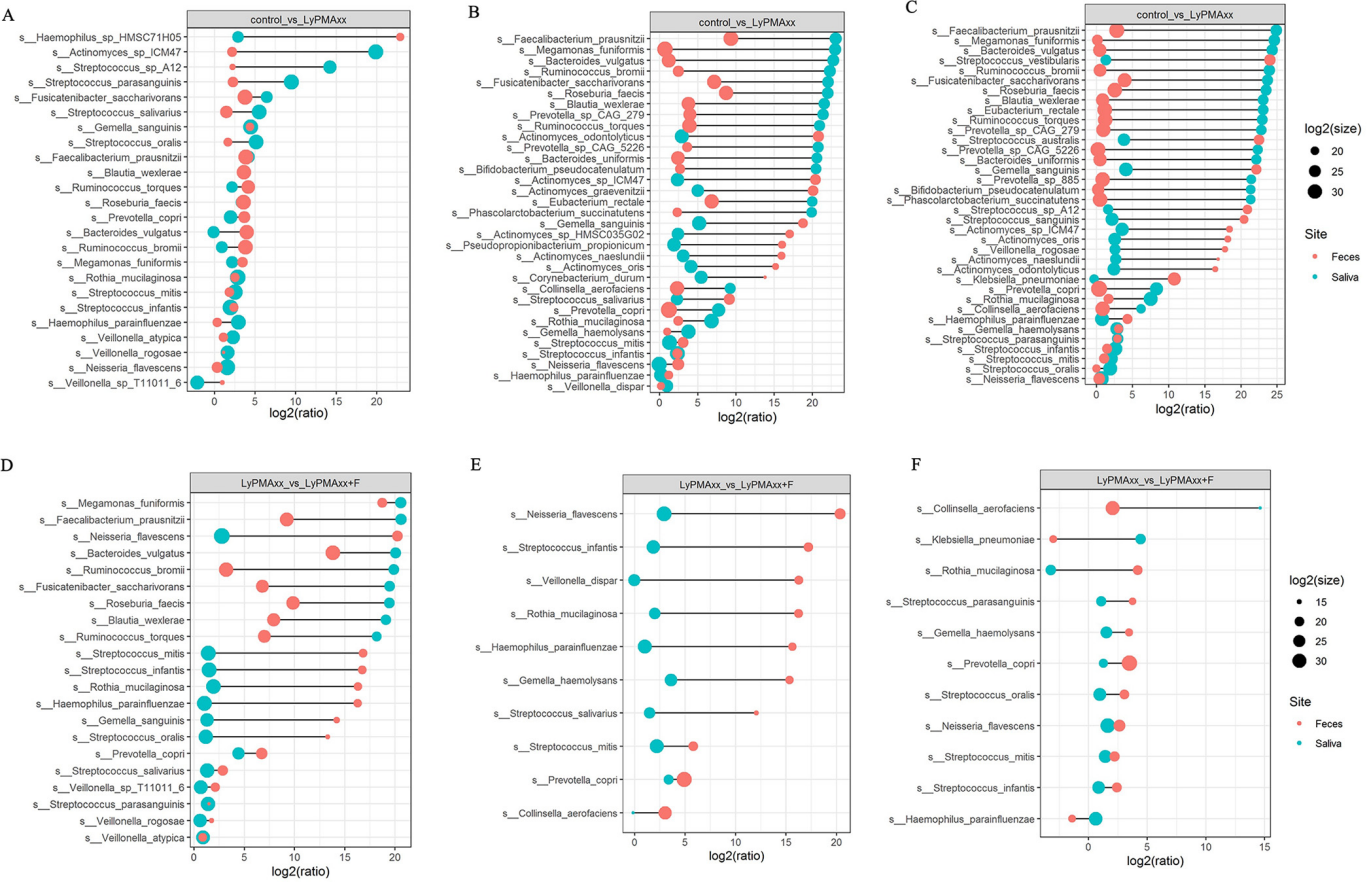
Effect of lyPMAxx and freezing on the shared species in the different sample types. (A to C) Fold changes in the absolute abundance of the shared species between saliva and feces by lyPMAxx in host 1 (A), host 2 (B), and host 3 (C). (D to F) Fold changes in the absolute abundance of the shared species between saliva and feces by freezing in host 1 (D), host 2 (E), and host 3 (F). Circle size represents the magnitude of absolute abundance. Feces samples are marked with orange circles, and saliva samples are marked with green circles.

The impact of lyPMAxx on the bacterial species depended on sample types (saliva and feces). The species that underwent the largest fold changes were Faecalibacterium prausnitzii, Megamonas funiformis, and Bacteroides vulgatus in saliva (Fig. 5A) and Gemella sanguinis and Collinsella aerofaciens in feces (Fig. 5B). The responses of different species to lyPMAxx in the saliva and fecal samples were next compared in each individual. In hosts 2 and 3, the majority of the shared species which were highly injured/dead in saliva showed a high intact/live rate in feces (Fig. 6B and C). These bacterial species were almost colon-dominant species, which were also found in saliva with low relative abundance. For example, Faecalibacterium prausnitzii, Megamonas funiformis, and Bacteroides vulgatus showed a higher than 4-fold reduction in the absolute abundance in saliva but showed no significant difference in feces (Fig. 5A and B and Fig. 6C). However, the situation was different in host 1, in which most of the shared species were highly alive in both sample types (Fig. 6A). These data indicate that both sample types and individual differences affected the response of microbial species to lyPMAxx treatment.

### Evaluation of frozen injury to the human microbial communities using the lyPMAxx method.

Frozen storage harms microbial vitality, but to the extent and the sensitivity of different bacteria remain elusive. We used lyPMAxx treatment to evaluate the impact of freezing on the microbiome community. The total microbial loads were significantly reduced in frozen samples compared to fresh samples in both saliva and feces. The living microbial loads in frozen saliva samples decreased to 27.5%, 28.9%, and 50% of that in the control group in the three hosts, respectively (Fig. 4A); in fecal samples, it decreased to 3.3%, 2.8%, and 10.8% (Fig. 4B). Freezing injured 65% and 94% of the live microbes in saliva and feces, respectively, suggesting greater damage to the fecal microbiome than the salivary microbiome.

The live microbiota composition changed after freezing in both saliva and fecal samples. At the phylum level, the relative abundance of *Bacteroidetes* was increased and that of *Proteobacteria* was reduced in the saliva of the three hosts after freezing (Fig. 4C). The relative abundance of *Actinobacteria* increased in frozen feces compared to that in fresh samples in the three hosts. *Bacteroidetes* was reduced to zero in host 1 feces without obvious changes in hosts 2 and 3 (Fig. 4D). We investigated the alpha diversity between samples before and after freezing. The alpha diversity was significantly increased in all saliva samples (Fig. 4E). However, it changed differently in the feces of the three hosts, which was reduced in host 1 and unchanged in hosts 2 and 3 (Fig. 4F). A PCoA plot based on the Bray-Curtis distance showed that the frozen samples separated from the fresh samples in each individual, indicating a significant effect of freezing on the microbiota composition (Fig. 4G and H). The Bray-Curtis distance between groups of intraindividual samples and interindividual samples indicated that frozen storage affected the microbiota, and the influence was smaller than individual differences in all saliva and feces samples (Fig. S3).

We next compared the absolute abundance of each species between fresh and frozen samples in different individuals and sample types. Among the 102 and 63 shared species in the saliva and feces of the three hosts, 82 and 53 of them significantly decreased after freeze-thaw treatment (Table S5). We presented the species with higher than 10-fold changes in saliva and 25-fold changes in feces in Fig. 5B and D as “freezing-responsive” species. In saliva, *Actinobaculum* sp. oral taxon 183 was the species most affected by freezing among the three hosts (Fig. 5B). In the feces of the three hosts, species belonging to *Bacteroidetes* and *Firmicutes* decreased significantly after freeze-thaw treatment, which were the main butyrate producers in the colon and were reported to regulate immunity (39, 40). Asaccharobacter celatus was relatively resistant to freezing, and its abundance decreased less than 2.5 times (Fig. 5D).

The freezing-responsive species were different between saliva and feces samples, in which most of these species belong to the phyla *Proteobacteria* and *Actinobacteria* in saliva and *Bacteroidetes* and *Firmicutes* in feces (Fig. 5B and D). In each individual, shared species between saliva and feces respond differently to freezing (Fig. 6D to F). Freezing injures microbial species depending on the different sample habitats.

## DISCUSSION

High-throughput sequencing is increasingly used to identify human microbiome-like salivary and fecal microbiota without clarifying whether the associated microbes are alive or dead (27, 28). In this study, we comprehensively explored the efficiency of lyPMAxx in host cell DNA removal and in distinguishing live and dead bacteria in both mock community and spiked human saliva and feces with absolute quantification. LyPMAxx removed more than 99% of the host cell and dead bacterial DNA and did not affect the live bacteria at a concentration of 50 to 130 μM in the simple community.

We evaluated the effect of lyPMAxx in saliva and feces samples by spiking-in four exogenous bacterial strains and found that lyPMAxx effectively inhibited the amplification of the host and dead bacterial DNA in saliva and feces. In our study, saliva samples contained up to 84% host DNA, which was depleted to 0.64% after lyPMAxx treatment. LyPMAxx removed 99% and 95% of the spike-in dead E. coli in saliva and feces, respectively. High host DNA contamination in saliva did not influence the effect of lyPMAxx on dead bacterial DNA here. Previous studies reported the function of PMA in biological matrices, showing incomplete removal of dead bacterial and free DNA. Wang et al. assessed the efficiency of 50 μM PMA in saliva and found incomplete elimination of relic DNA (23). Mancabelli et al. indicated that treating the saliva sample with 75 μM PMAxx resulted in a 73% reduction in the sequencing reads corresponding to the added free DNA, and the percentage of the added free DNA observed in the fecal sample increased with PMAxx treatment (22). The efficiency of lyPMAxx in depleting spike-in dead bacteria of the human microbiome samples was higher that in than those reports. This may be due to several factors, including the total bacterial concentration, osmotic lysis treatment, incubation time, and light conditions (19). In our study, the fecal samples were diluted 45 times to avoid the influence of high suspended solids content and microbial biomass, which might affect the photoactive efficiency. This is consistent with the previous study, in which Papanicolas et al. reported that PMA treatment excluded >97% of nonviable cells in feces from amplification with appropriate sample dilution, without significantly affecting the amplification of DNA from viable cells (24). Osmotic lysis and PMAxx treatment were conducted in the sterile H_2_O, which might also elevate the PMAxx efficiency. The photoreactive DNA-binding process was implemented under an LED blue light (470 nm, 60 W) for 15 min following the manufacturer’s instructions. Taking these findings together, the working conditions of PMA need to be optimized based on the levels of suspended solids and microbial biomass. Meanwhile, sample size also affected the accuracy of the results. For cost consideration, we took samples from three hosts for analysis. Though the sample size was limited, when evaluating the efficiency of LyPMAxx on the spike-in live/dead bacterial strains, the efficacy was relatively consistent among the three volunteers (Table S3).

We found that lyPMAxx also affected the spike-in live E. faecalis (*C_T_* value increased by 0.88 ± 0.37 and 0.97 ± 0.78 in saliva and feces, respectively) and *S. enterica* (*C_T_* value increased by 0.38 ± 0.19 and 0.37 ± 0.50 in saliva and feces, respectively) (Fig. 2B and D). LyPMAxx treatment exhibits a bigger influence on E. faecalis than *S. enterica*. We suppose that it might be due to species preference of PMAxx penetrating living cells. Theoretically, the membrane-impermeant dye is highly selective in penetrating only dead/injured bacterial cells; however, it may also be taken up by live cells depending on the bacterial species and dye concentration (41, 42). After lyPMAxx treatment, the dead-to-live ratios significantly changed, but the change of the ratios between live microbes (ratio of E. faecalis to S. enterica changed from 1.77 ± 0.39 to 1.05 ± 0.40 before and after lyPMAxx in saliva and from 3.33 ± 0.31 to 2.05 ± 0.49 in feces) were small (Fig. 4C and 4D). When considering the quantitative ability of lyPMAxx-metagenomic sequencing on viable microbiota, we think that it is a relatively feasible method based on our results, given that metagenomic sequencing itself is not an accurate approach owing to the DNA extraction bias, sequencing, and bioinformatics classification accuracy (38).

The bacterial load decreased to 40.3% and 36.5% in fresh saliva and fecal samples, respectively, after LyPMAxx treatment in our study (Fig. 4A and B). It was reported that the intact cells accounted for 49% of the fecal bacteria by flow cytometry (3). The live bacteria in saliva could range from nearly 0% up to 100% throughout a typical day (43). Alpha diversity was significantly reduced in both fresh saliva and feces after lyPMAxx treatment (Fig. 4E and F). This is consistent with a previous report, in which viable diversity (lyPMAxx-treated group) was significantly lower than diversity observed in control fecal specimens (26). The salivary and fecal microbiota alpha diversities were all significantly decreased by lyPMAxx treatment for all the three hosts (Fig. 4E). Bray-Curtis distance analysis showed that lyPMAxx affected the microbiota, but the influence was smaller than individual differences across three hosts (Fig. S3). The proportion of *Proteobacteria* was increased, while that of *Firmicutes* and *Actinobacteria* decreased in saliva of the three hosts (Fig. 4C). In feces, the relative abundance of *Firmicutes* was reduced, and that of *Bacteroidetes* was increased (Fig. 4D). Through exploring the effect of lyPMAxx on the microbiota in different persons and sample types, we found that the microbial species respond to lyPMAxx depending on individual differences and sample habitat (Fig. 5 and 6). Wang et al. also reported that the abundance changes of the taxa by PMA treatment varied greatly in different samples (23).

Even though the application of PMA in discriminating live from dead bacteria had been widely used, standardized working conditions and treatment procedures were lacking, especially in complex microbiome samples. Compared to previous peer-reviewed studies, we made the following modifications to evaluate the efficiency of lyPMAxx sequencing more comprehensively: (i) Wang et al. evaluated the efficiency of PMA-16S rRNA amplicon sequencing in human saliva with spike-in E. coli. They spiked in live/dead E. coli cells at a ratio of 1:1, and E. coli existed in most of the nonspiked native samples. We spiked in four strains, including two live/dead Gram+ bacteria and two live/dead Gram– bacteria, three and four of which were absent in the native fecal and salivary samples (Table S2), which would be more conducive to the quantification of the live and dead spike-in bacteria. (ii) Some bacteria were affected by PMAxx but could not be quantified with relative abundance change because the total microbial load was reduced significantly. To clarify the effect of lyPMAxx among different sample types and individuals, absolute abundance was calculated taking account of the difference in bacterial loads here. (iii) We combined osmotic lysis with PMAxx-metagenomic sequencing and depleted the influence of host DNA in high host contamination samples such as saliva. (iv) To evaluate the stability of lyPMAxx, three reduplications were carried out in our research. The function of lyPMAxx was very stable in the reduplicated samples. Meanwhile, we found that the DNA extraction efficiency of different microbes biased the quantification of the bacteria significantly (Fig. S1, Text S1). The DNA extraction efficiency of *L. plantarum* (G+) was very low in our research, and this was consistent with the previous study (38). When further exploring the susceptibility of different bacterial species to lyPMAxx and freezing among different persons, we found that the response of microbial species to lyPMAxx and freezing varied across hosts (Fig. 5). Thus, expanding the sample size is necessary to study the susceptibility of different bacterial species to lyPMAxx and freezing among people.

Freeze-thaw storage affected the cell membrane integrity of the microbes, which may reduce the culturable bacteria and the therapeutic effect of FMT (44). With the lyPMAxx method, we found that a freeze-thaw cycle killed/injured 65% and 94% of the viable bacteria in saliva and feces, respectively. The impact of freezing storage on the fecal microbiota was greater than that on the salivary microbes. The alpha diversities of salivary microbiota were elevated by freezing in all three hosts; however, fecal microbiota alpha diversity changed in different directions in the hosts (Fig. 4E and F). Bray-Curtis distance analysis indicated that frozen storage affected the microbiota structure, and the influence was smaller than individual differences in all saliva and feces samples (Fig. S3). The relative abundance of *Bacteroidetes* was increased, and that of *Proteobacteria* was reduced in the saliva of the three hosts after freezing in our study (Fig. 4C). The relative abundance of *Actinobacteria* increased in frozen feces (Fig. 4D). Dorsaz et al. reported that the relative abundance of *Bacteroidetes* decreased and that of *Firmicutes* was not much affected in feces by freezing with DNase pretreatment and 16S rRNA gene amplicon sequencing (25). Takahashi et al. claimed that −20°C freeze-thawing did not significantly affect the fecal bacterial structure with PMA-16S rRNA amplicon sequencing, but the recovery effect of FMT using frozen feces was reduced in a freezing time-dependent manner (45). However, they focused on the resulting “viable” communities without evaluating the efficiency of their methods in depleting relic DNA in those studies, and they studied the effect of freezing on fecal microbiome composition but ignored the variation of the overall microbial loads. Papanicolas et al. concluded that freeze-thaw did not alter viable microbiota composition, while it reduced overall levels of viable bacteria significantly (26). Therefore, fresh samples or better protectants will be needed to protect the overall microbiota and some freezing-sensitive microbes.

### Conclusions.

We systematically evaluated the efficacy of lyPMAxx in host and dead bacterial DNA removal and its effect on live bacteria in human microbiome samples by spiking-in four representative strains. LyPMAxx could effectively deplete host and dead bacterial DNA with limited influence on live microbes. Osmotic lysis and PMAxx treatment are simple to implement and low cost. Optimizing the procedure of PMA-sequencing is important for functional studies of the human microbiome. LyPMAxx and freezing treatment reduced the total bacterial load significantly. By comparing the absolute abundances of the bacterium taxa among different sample types and hosts, the bacterial species were found to respond to lyPMAxx/freezing depending on sample habitat and individual differences. LyPMAxx could effectively deplete host contamination and discriminate between live and dead bacteria in human saliva and feces samples in our study.

## MATERIALS AND METHODS

### Materials.

Caco2 cells (human colonic adenocarcinoma cell line, HTB-37) were purchased from ATCC (Rockville, MD, USA). Escherichia coli K12 was purchased from Sangon Biotech (Shanghai, China). Salmonella enterica subsp. *enterica* (ATCC14028) and Enterococcus faecalis (ATCC29212) were purchased from HB-CICC (Wuhan, Hubei, China). Lactobacillus plantarum R1012 came from Lallemand (Toulouse, France).

### Caco2 cells culture and count.

Caco2 cells were cultured in Dulbecco’s modified Eagle’s medium (DMEM) supplemented with 10% fetal bovine serum (FBS; Excell Biological Technology, Shanghai, China) and 1% penicillin-streptomycin. All cells were incubated in T75 flasks at 37°C in a humidified incubator with 5% CO_2_, and the medium was replaced every 2 days. Cells were passaged (1:1) with 0.25% trypsin-EDTA when the cells reached 80% confluence. Cells between 16 and 32 generations were used for all experiments. The number of Caco2 cells was determined using the JIMBIO FIL counter (Jimbio, Changzhou, China).

### Bacterial culture and count.

Activated E. coli and S. enterica were inoculated into 10 mL Luria-Bertani (LB) medium and incubated at 37°C with 180-rpm shaking to reach log-phase growth (optical density at 600 nm [OD_600_], 0.680 for E. coli; OD_600_, 0.230 for S. enterica). *L. plantarum* and E. faecalis were inoculated into 10 mL MRS medium at 37°C to reach log-phase growth (OD_600_, 1.260 for *L. plantarum*; OD_600_, 0.620 for E. faecalis). The viable counts were obtained by plate counting and flow cytometry (see Tables S6 and S7 at https://doi.org/10.6084/m9.figshare.21982448.v1 and https://doi.org/10.6084/m9.figshare.21670967.v1, Fig. S4).

### Preparation of simple synthetic community.

We constructed a simple synthetic community, comprising two Gram-negative bacteria (heat-killed E. coli and live S. enterica), two Gram-positive bacteria (heat-killed *L. plantarum* and live E. faecalis), and a human cell line Caco2 representing host cells (Table 1). The dead bacteria were killed by heating at 96°C for 15 min (E. coli) or 20 min (*L. plantarum*). The bacterial activity was confirmed via plate counting and flow cytometry (propidium iodide and SYTO 9).

### Saliva and feces collection, pretreatment, spike-in culture.

Three volunteers aged 25 to 30 years, without a history of antibiotic usage within 3 months, were recruited. Saliva and feces samples of the volunteers were collected on the same day. Volunteers were asked to fast for 1 h before saliva sample collection. A total of 5 mL of unstimulated saliva was collected into sterile conical tubes.

Feces were collected from the same volunteers using a sterile stool sampler and processed immediately under anaerobic conditions (Coy-01, Michigan, USA). Fresh feces (150 mg) were collected into a 50-mL sterile centrifuge tube. Fresh feces were diluted 45-fold in phosphate-buffered saline (PBS; containing 0.05% l-cysteine hydrochloride) and added with 1-mm sterile grinding beads. The diluted fecal samples were blended twice with a tissue grinder (Servicebio KZ-11, Wuhan, China) at 25 Hz for 2 min to homogenize the fecal pellet.

Saliva or diluted fecal samples (500 μL) without spiking-in bacteria were used to determine whether four spike-in bacteria existed in native saliva or feces by qPCR. Then, 4.5-mL saliva or diluted fecal samples were spiked with exogenous bacteria and divided into 9 equal aliquots. Every 3 aliquots were used in control group (without lyPMAxx treatment), lyPMAxx treatment group, and freezing plus lyPMAxx treatment group. The amount of the spike-in bacteria was determined with 16S rRNA plasmid-based absolute quantification (34) to about 5 to 15% of the total bacteria to avoid affecting the detection of the endogenous microbiota. Sterile PBS was used as the negative control, and the spike-in bacteria were used as the positive control.

For frozen samples, three aliquots of spiked salivary and fecal samples were added with glycerol to a final concentration of 20% and then frozen at −80°C for more than 72 h. After freezing, the samples were thawed (fecal samples were thawed and conducted under anaerobic conditions), treated with lyPMAxx, and extracted DNA.

### Osmotic lysis of host cells and PMAxx treatment.

We constructed a simple synthetic community as described earlier to validate the work conditions of lyPMAxx. The treatment procedures of lyPMAxx are shown in Fig. 1A. The synthetic community (500 μL) was centrifuged at 14,000 rpm for 15 min. The supernatant was discarded, and the cell pellet was resuspended in 500 μL sterile H_2_O. After a brief vortexing, the samples were placed at room temperature for 5 min to osmotically lyse the human cells. Then, 20 mM PMAxx (Biotium, CA, USA) was diluted in sterile water to a 2 mM stock solution. The samples were added at 2.7 μL, 13.4 μL, 24.1 μL, and 34.8 μL of the 2 mM PMAxx stock solution and complemented with sterile water to 534.8 μL to make the final volume consistent (i.e., final concentrations of 10, 50, 90, and 130 μM PMAxx). The samples were incubated in the dark at room temperature for 10 min. Samples were then laid horizontally <20 cm under an LED blue light (470 nm, 60 W) for 15 min, with gentle manual inverting and shaking of the tubes every 5 min. After exposure, samples were frozen at −80°C until DNA extraction.

Spiked-in saliva and fecal samples were osmotically lysed and treated with PMAxx similarly to the synthetic community as shown in Fig. 1B; 50 μM PMAxx was used in saliva. High turbidity and microbial biomass content in feces may discount the effectivity of PMAxx (19), and PMAxx did not show any toxicity on live bacteria at a concentration of 130 μM (Fig. 2A). Thus, we applied 130 μM PMAxx in feces.

### DNA extraction.

After lyPMAxx treatment, DNA was extracted, followed by qPCR and shotgun metagenomic sequencing. To measure the repeatability of lyPMAxx-sequencing, three replicates were conducted in each treatment group for each individual. DNA was extracted using the PowerFecal Pro DNA kit (QIAamp, MO BIO Laboratories, Carlsbad, CA, USA) in accordance with the manufacturer’s instructions. DNA was eluted in 50-μL Qiagen elution buffer with concentrations quantitated using a nanodrop device (Thermo, USA).

### Real-time PCR assay.

Five pairs of special primers were synthesized for different target cells (Table 2): Caco2, E. coli, S. enterica, E. faecalis, and *L. plantarum*. A pair of bacterial universal primers in 16S rRNA gene V6 regions was used to quantify the total amount of the bacteria. qPCR was conducted on a CFX Connect real-time system (BIO-RAD, California, USA) with a TB green premix *Ex Taq* II (TaKaRa, Osaka, Japan). Each qPCR volume totaled 25 μL, containing 12.5 μL TB green premix Ex Taq, 1 μL DNA, 1 μM forward and reverse primers, and the remainder of the water. The cycling conditions included an initial 30-s hot start at 95°C, followed by 40 cycles of 5 s at 95°C, 30 s at 60°C, and a final extension step (10 s at 95°C). All reactions were performed in triplicate.

**TABLE 2:** Primers used in this study

Cell	Target	Sequence^*a*^	Amplicon length (bp)	Reference
Bacteria	16S rRNA V6	F: AAACTCAAAKGAATTGACGG R: CTCACRRCACGAGCTGAC	136	46
Caco2	ZO-1	F: TTCACGCAGTTACGAGCAAG R: TTGGTGTTTGAAGGCAGAGC	141	47
*E. faecalis* ATCC 29212	16S rRNA	F: CGCTTCTTTCCTCCCGAGT R: GCCATGCGGCATAAACTG	143	48
*E.coli K12*	*uidA*	F: CGGAAGCAACGCGTAAACTC R: TGAGCGTCGCAGAACATTACA	90	49
S. enterica ATCC14028	InvA	F: GCTGCTTTCTCTACTTAAC R: GTAATGGAATGACGAACAT	95	50
*L. plantarum* R1012	LPrecA	F:GTGGTGCGGTCGATATTTTAGTT R: TCAGCCGCGCTTGTAACC	108	51

a F, forward; R, reverse.

### Metagenomic sequencing.

Sequencing libraries of salivary and fecal DNA were generated using a NxSeq Ultra low-DNA library kit (Lucigen, Middleton, WI, USA). The library quality was assessed with a Qubit 4.0 fluorometer (Life Technologies, Grand Island, NY) and Qsep400 high-throughput nucleic acid protein analysis system (Houze Biological Technology, Hangzhou, China). Then it was sequenced on an Illumina NovaSeq 6000 platform, and 150-bp paired-end reads were generated.

### Microbiota load and absolute abundance calculation.

Standard curves of the 16S rRNA gene were built based on a plasmid containing the total 16S rRNA gene sequence of E. coli with qPCR. The 16S rRNA gene copy numbers were calculated as described previously (21, 30). Microbiota load was defined as the total 16S rRNA copy numbers per mL of saliva or g of feces. We then utilized this measurement of microbiota load to compute the absolute abundances of microbial taxa by scaling the relative abundances of microbes in a sample by the microbiota load of that sample.

### Bioinformatic analysis of shotgun metagenomic data.

Raw metagenomic shotgun reads were quality filtered using fastp (version 0.21.0) (31). All reads were aligned to the human genome to determine the ratio of host reads using Bowtie 2 (version 2.4.2) (32). In the spiked-in complex samples, the proportions of human reads were calculated after removing the reads of the spike-in bacteria. The host reads were all removed from downstream analyses. MetaPhlAn3 (version 3.0.10) (33) was used to produce a taxon table. Further bioinformatics analyses, data statistics, and visualization were performed in R (version 4.0.0) using vegan (34), ggplot2 (35), and pheatmap (36). Alpha diversity was calculated using vegan (34). Principal-coordinate analysis (PCoA) was performed using Bray-Curtis dissimilarity based on the relative abundances and visualized via Quantitative Insights into Microbial Ecology 2 (QIIME 2) (37). The ratio of the absolute abundances between samples was calculated with a paired Wilcoxon test.

### Statistical analysis.

Statistical analysis was performed with GraphPad Prism 8.21 software (La Jolla, CA, USA). Significant differences were identified with paired *t* tests for total 16S rRNA gene copy numbers in saliva and feces and unpaired *t* tests for DNA extraction efficiency of several strains. The unpaired Wilcoxon test was used for alpha diversity of the saliva and feces microbiota, and the paired Wilcoxon test was used for lyPMAxx and freezing effect on the shared species in different individuals. *, *P* < 0.05; **, *P* < 0.01; ***, *P* < 0.001; ****, *P* < 0.0001. The values were expressed as the means ± the standard error of the mean (SEM).

### Ethics approval.

The human trials were approved by the Ethical Committees of Affiliated Stomatological Hospital of Nanchang University (ethical approval no. ET2021023). Written informed consent was obtained from all subjects.

### Data and material availability.

The sequencing data were deposited at NGDC (the National Genomics Data Center) with project number PRJCA010533. All data relevant to the study are included in the article or uploaded as supplemental information.

10.1128/msystems.00738-22.1 TEXT S1 (1) DNA extraction efficiency influences the results of microbiome analysis. (2) Quantification of the four bacteria in the simple synthetic community, native or spiked-in saliva and feces samples, with qPCR. (3) Preparation and confirmation of the high percentage of dead spike-in bacteria. (4) Flow cytometry for the detection of the live/dead spike-in bacteria. (5) The death rates of the salivary and fecal bacterial species were associated with human health. (6) The association of the freezing-responsive and Freezing-resilient species to human health. Download Text S1, DOCX file, 0.05 MB. Copyright © 2023 Liu et al. 2023 Liu et al. https://creativecommons.org/licenses/by/4.0/ This content is distributed under the terms of the Creative Commons Attribution 4.0 International license.

10.1128/msystems.00738-22.5 FIG S4 Live/dead bacterial detection with flow cytometry. Propidium iodide (PI) and SYTO9 staining showed the live/dead bacteria before and after heat treatment. PI^+^ cells, PI^+^SYTO9^+^ cells, and SYTO9^+^ cells were considered dead, injured, and live cells, respectively. (A) E. coli in the logarithmic growth phase before heat treatment; (B) E. coli after heat treatment; (C) E. faecalis in the logarithmic growth phase before heat treatment; (D) E. faecalis after heat treatment; (E) *L. plantarum* in the logarithmic growth phase before heat treatment; (F) *L. plantarum* after heat treatment; (G) S. enterica in the logarithmic growth phase before heat treatment; (H) S. enterica after heat treatment. Download FIG S4, TIF file, 0.9 MB. Copyright © 2023 Liu et al. 2023 Liu et al. https://creativecommons.org/licenses/by/4.0/ This content is distributed under the terms of the Creative Commons Attribution 4.0 International license.
