# Supplementary material for: Systematic Evaluation of the Viable Microbiome in the Human Oral and Gut Samples with Spike-in Gram+/– Bacteria
Source: mSystems. 2023 Mar 27;8(2):e00738-22. doi: 10.1128/msystems.00738-22 (PMC10134872; doi:10.1128/msystems.00738-22)
Supplement: TABLE S1 [file msystems.00738-22-s0001.docx]

| Number | Sample | DNA  (ng/μl) | PMAxx  (μM) | Ct (Caco2) | | Ct (*E.coli* K12, dead) | | Ct (*L. plantarum* R1012*,* dead) | | Ct (S.enteria ATCC14028, live) | | Ct (S.faecalis ATCC29212, live) | |
| --- | --- | --- | --- | --- | --- | --- | --- | --- | --- | --- | --- | --- | --- |
| 1 | Negative control | -0.3 | 0 | 37.42 | 37.84 | N/A | 38.28 | N/A | 36.75 | 34.82 | 37.83 | 34.81 | 35.45 |
| 2 | Mixture | 41.7 | 0 | 26.16 | 26.13 | 20.09 | 20.05 | 25.27 | 25.27 | 16.29 | 15.99 | 13.80 | 13.81 |
| 3 | Mixture | 33.4 | 10 | 27.61 | 28.16 | 24.35 | 24.27 | 32.02 | 31.23 | 16.12 | 16.22 | 13.71 | 13.83 |
| 4 | Mixture | 27.6 | 50 | 33.92 | 34.41 | 29.72 | 30.30 | 33.74 | 33.27 | 15.75 | 15.65 | 13.41 | 13.42 |
| 5 | Mixture | 26.5 | 90 | 37.88 | N/A | 32.64 | 32.65 | 34.74 | 35.24 | 16.03 | 16.17 | 13.78 | 13.62 |
| 6 | Mixture | 19.4 | 130 | N/A | N/A | 34.45 | 34.19 | 35.35 | 36.14 | 16.59 | 16.69 | 14.12 | 14.06 |
