# Supplementary figures and images for: Systematic Evaluation of the Viable Microbiome in the Human Oral and Gut Samples with Spike-in Gram+/– Bacteria
Source: mSystems. 2023 Mar 27;8(2):e00738-22. doi: 10.1128/msystems.00738-22 (PMC10134872; doi:10.1128/msystems.00738-22)

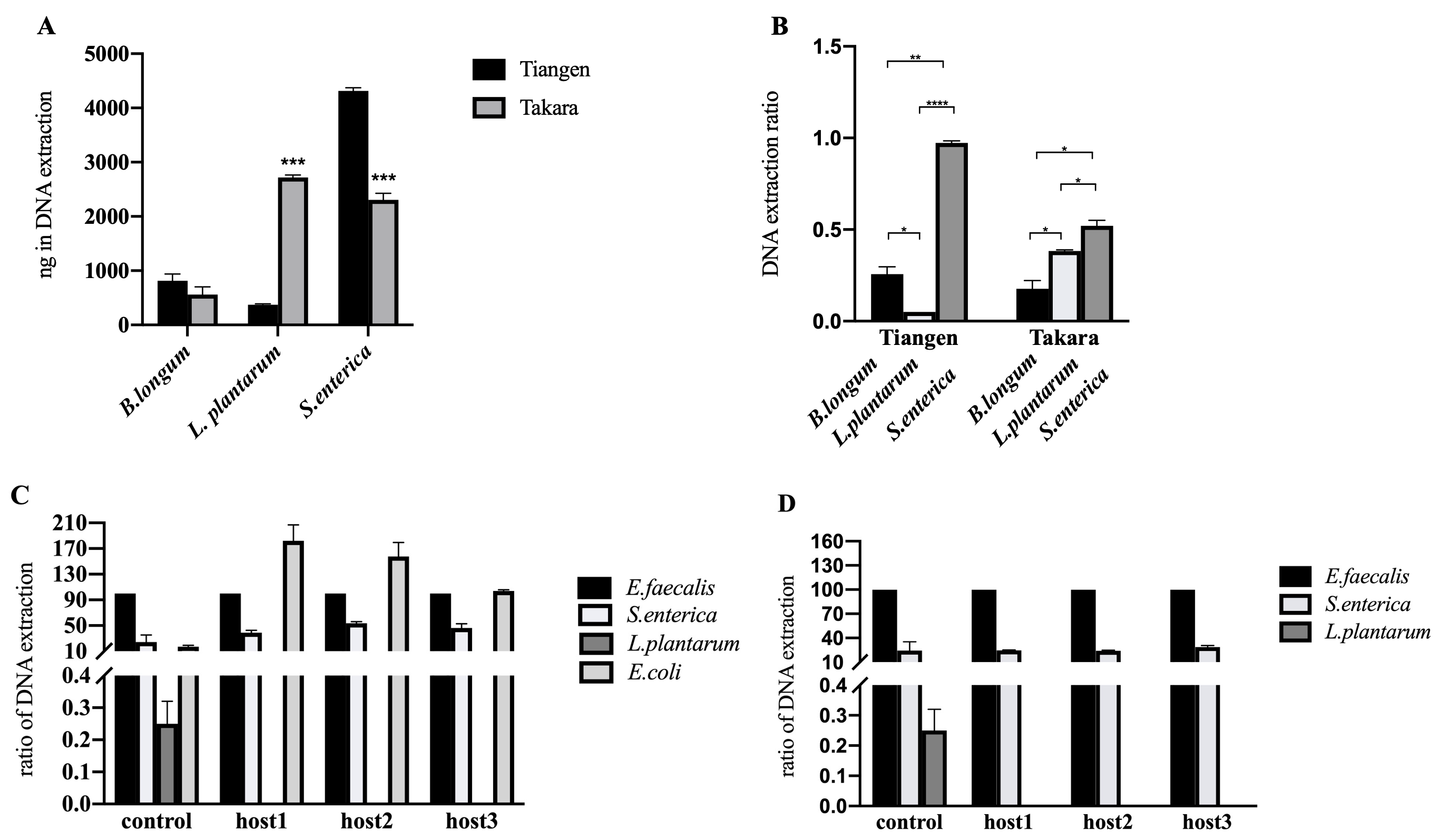

Supplement: FIG S1 [file msystems.00738-22-s0004.tif]

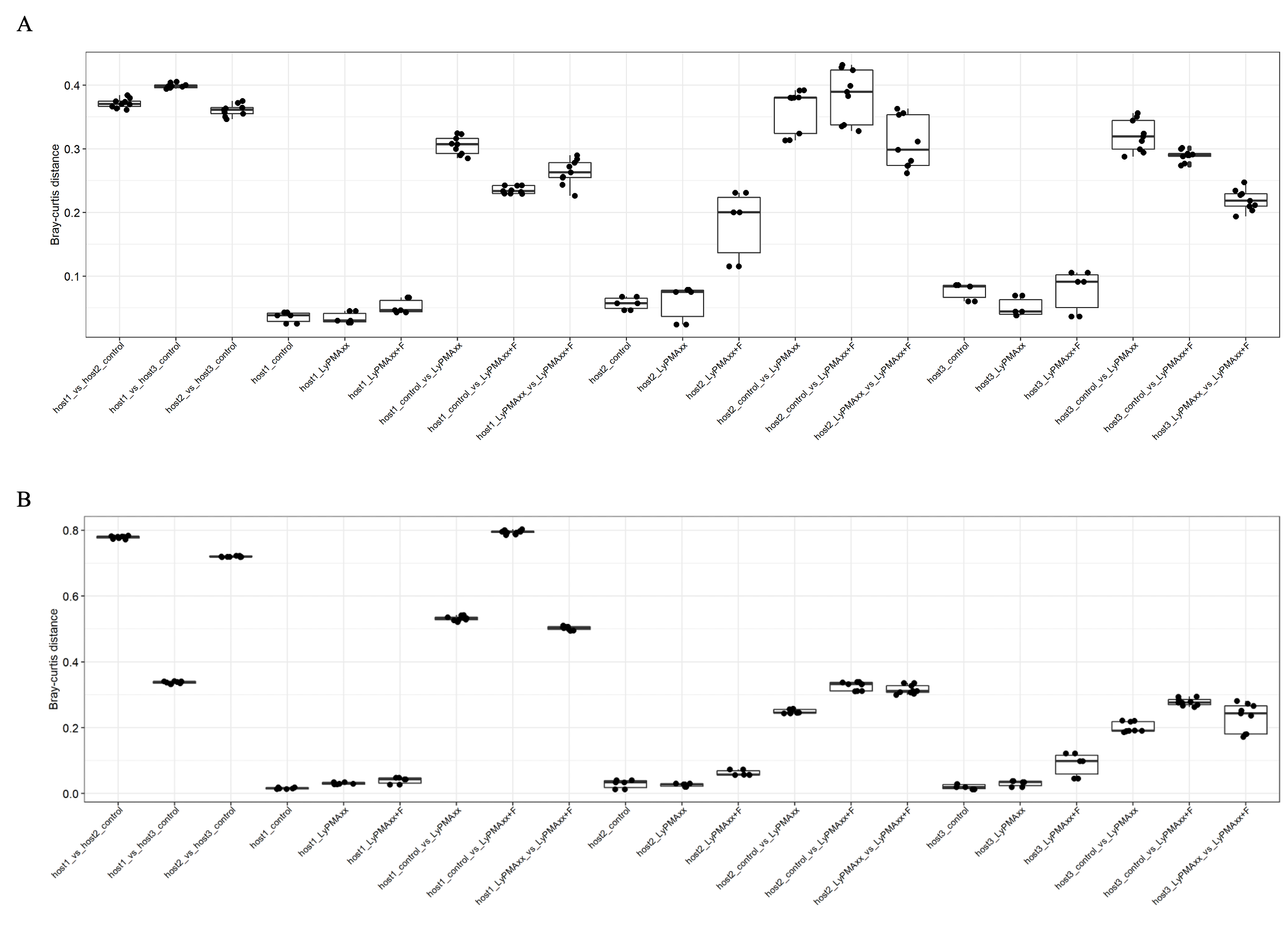

Supplement: FIG S3 [file msystems.00738-22-s0005.tif]

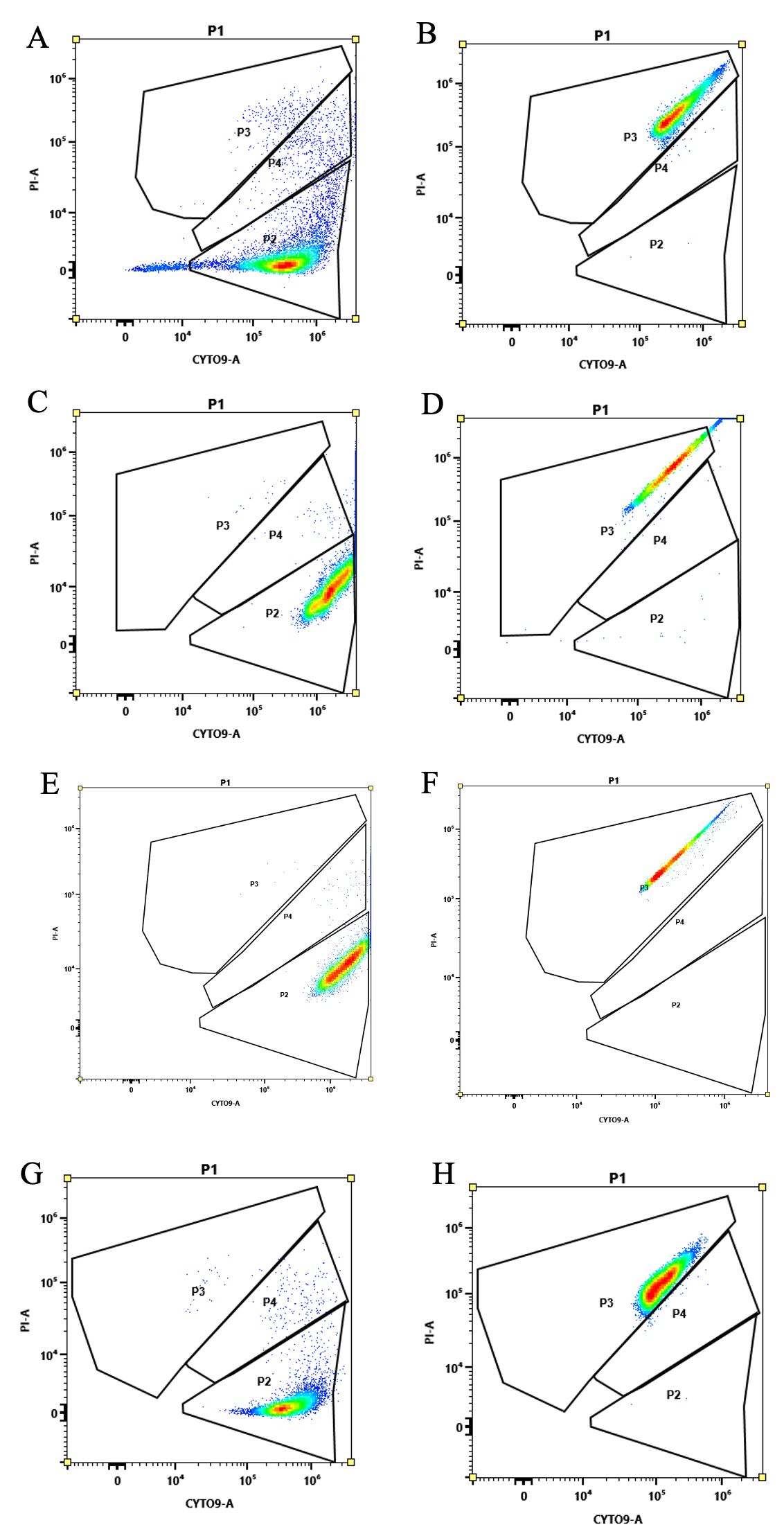

Supplement: FIG S4 [file msystems.00738-22-s0006.tif]
