# Supplementary material for: Systematic Evaluation of the Viable Microbiome in the Human Oral and Gut Samples with Spike-in Gram+/– Bacteria
Source: mSystems. 2023 Mar 27;8(2):e00738-22. doi: 10.1128/msystems.00738-22 (PMC10134872; doi:10.1128/msystems.00738-22)
Supplement: TABLE S2 [file msystems.00738-22-s0008.docx]

| Strain | Saliva or Feces | Ct (host 1) | | Ct (host 2) | | Ct (host 3) | | Ct (Negative control) | |
| --- | --- | --- | --- | --- | --- | --- | --- | --- | --- |
| *E. coli* K12 | saliva | 36.60 | 35.71 | N/A | N/A | N/A | N/A | 36.93 | N/A |
|  | feces | 34.95 | 34.42 | 28.20 | 28.11 | 28.61 | 28.53 | N/A | N/A |
| *L. plantarum* R1012 | saliva | 35.09 | 34.58 | N/A | N/A | N/A | N/A | 36.19 | 34.78 |
|  | feces | N/A | N/A | N/A | N/A | N/A | N/A | N/A | N/A |
| *S. enterica* ATCC14028 | saliva | 35.99 | 37.81 | N/A | N/A | N/A | N/A | 36.02 | 36.31 |
|  | feces | N/A | N/A | N/A | N/A | N/A | N/A | N/A | N/A |
| *E. faecalis* ATCC29212 | saliva | 32.56 | 32.45 | N/A | N/A | N/A | N/A | 36.11 | 34.05 |
|  | feces | N/A | N/A | N/A | N/A | 32.32 | 32.24 | N/A | N/A |
