# Supplementary material for: Systematic Evaluation of the Viable Microbiome in the Human Oral and Gut Samples with Spike-in Gram+/– Bacteria
Source: mSystems. 2023 Mar 27;8(2):e00738-22. doi: 10.1128/msystems.00738-22 (PMC10134872; doi:10.1128/msystems.00738-22)
Supplement: TABLE S3 [file msystems.00738-22-s0009.docx]

| Strain | Saliva or Feces | Group | Host1 | Host2 | Host3 |
| --- | --- | --- | --- | --- | --- |
| E. coli K12 (dead) | saliva | control | 24.30±0.16 | 23.96±0.14 | 24.65±0.05 |
|  |  | lyPMAxx | 37.00±0.59 | 35.05±2.80 | 35.99±0.62 |
| *L. plantarum* R1012 (dead) | saliva | control | 29.10±0.43 | 28.28±0.11 | 30.01±0.17 |
|  |  | lyPMAxx | 36.29±0.08 | 35.45±1.24 | 36.35±0. 35 |
|  | feces | control | N/A | N/A | N/A |
|  |  | lyPMAxx | N/A | N/A | N/A |
| *S. enterica* ATCC14028 (live) | saliva | control | 23.02±0.58 | 22.47±0.25 | 22.37±0.24 |
|  |  | lyPMAxx | 22.99±0.16 | 22.01±0.02 | 22.48±0.23 |
|  | feces | control | 25.57±0.19 | 25.98±0.33 | 26.01±0.21 |
|  |  | lyPMAxx | 25.47±0.63 | 26.62±0.55 | 26.20±0.44 |
| *E. faecalis* ATCC29212 (live) | saliva | control | 19.34±0.06 | 19.07±0.18 | 19.09±0.08 |
|  |  | lyPMAxx | 19.97±0.43 | 19.95±0.34 | 20.18±0.16 |
|  | feces | control | 21.91±0.28 | 18.57±0.22 | 22.58±0.17 |
|  |  | lyPMAxx | 22.60±0.63 | 20.38±0.31 | 22.89±0.66 |
