# Supplementary material for: Systematic Evaluation of the Viable Microbiome in the Human Oral and Gut Samples with Spike-in Gram+/– Bacteria
Source: mSystems. 2023 Mar 27;8(2):e00738-22. doi: 10.1128/msystems.00738-22 (PMC10134872; doi:10.1128/msystems.00738-22)
Supplement: TABLE S4 [file msystems.00738-22-s0002.docx]

| Sample | Ct (host 1) | | | Ct (host 2) | | | Ct (host 3) | | |
| --- | --- | --- | --- | --- | --- | --- | --- | --- | --- |
| Feces | 35.48 | 34.95 | 34.42 | 27.9 | 28.2 | 28.11 | 28.76 | 28.61 | 28.53 |
| Spike-in +feces （control） | 29.92 | 30.53 | 30.54 | 27.91 | 28.02 | 28.19 | 28.14 | 28.05 | 27.99 |
| Spike-in +feces (lyPMAxx) | 35.02 | 34.4 | 34.67 | 28.56 | 28.63 | 28.62 | 28.66 | 29.24 | 29.03 |
| Negative control | 37.82 | N/A | N/A | 37.82 | N/A | N/A | 37.82 | N/A | N/A |
