# Supplementary material for: Systematic Evaluation of the Viable Microbiome in the Human Oral and Gut Samples with Spike-in Gram+/– Bacteria
Source: mSystems. 2023 Mar 27;8(2):e00738-22. doi: 10.1128/msystems.00738-22 (PMC10134872; doi:10.1128/msystems.00738-22)
Supplement: TEXT S1 [file msystems.00738-22-s0007.docx]

Supplementary material

1. **DNA extraction efficiency influences the results of microbiome analysis**

By spiking-in different bacterial strains, we found that DNA extraction efficiency significantly influences the quantification of the microbes. Different DNA extraction kits were dramatically different in their capabilities of DNA extraction experiments. DNA was first extracted using TIANamp Stool DNA Kit (Tiangen, China) and MiniBEST Bacteria Genomic DNA Extraction Kit (Takara, Japan) following the manufacturer’s instructions. The bacterial numbers of each sample were the same as validated with plate counting. DNA concentrations of three different bacteria (*L. plantarum* (G^+^)*, S. enteria* (G^-^), and *B. longum* (G^+^)) were evaluated using the Qubit dsDNA BR Assay kit (Invitrogen). Distinct DNA quantities were obtained from the same bacterial numbers with different kits. The DNA amount of *L. plantarum* extracted with the Takara kit was seven times more than that of the Tiangen kit (Fig. S1A). The ratio of the DNA extraction quantity to the theoretical DNA yields (the number of cells multiplied by the amount of DNA per cell) was calculated and considered as extraction efficiency. The extraction efficiency of *S. enterica*, *B. longum,* and *L. plantarum* were 97.0 ± 1.2%, 26.0 ± 4.0%, 5.0 ± 0.3% by Tiangen kit and were 52.0 ± 3.0%, 18.0 ± 4.5%, 38.0 ± 0.6% by Takara kit (Fig. S1B). The extraction efficiency of *L.plantarum* by Tiangen kit was very low.

In spike-in control, saliva, and feces samples, the DNA extraction efficiency of PowerFecal Pro DNA Kit (QIAamp, MO BIO Laboratories, Carlsbad, CA, USA) on the four bacterial strains (*E. coli* (dead, G^-^), *L. plantarum* (dead, G^+^)*, S. enteria* (live, G^-^) and *E. faecali* (live, G^+^)) was estimated by calculating the ratio of the relative abundance of sequencing to the number of the spike-in bacteria. The DNA extraction ratio of each bacteria was normalized to that of *E. faecalis* and the DNA extraction ratio of *E. faecalis* was recognized as 100%. As shown in Fig. S1C, PowerFecal Pro DNA Kit exhibited different extraction efficiency on different bacteria. *E. faecalis* had the highest extraction efficiency in the spike-in control community. The extraction efficiency of *S. enterica*, *E. coli*, *L. plantarum* were 24.5 ± 10.9%, 16.9 ± 2.5%, and 0.3 ± 0.1% compared to that of *E. faecalis.* The extraction efficiency of *L.plantarum* was very low in the spike-in control, which was similar to that with TIANamp Stool DNA Kit (Fig. S1B). In spiked-in saliva and feces samples, the extraction efficiency of *L. plantarum* was decreased to zero (Fig. S1C and S1D). These results indicated that DNA extraction kits, bacterial strains, and sample types affected DNA extraction efficiencies.

1. **Quantification of the four bacteria in the simple synthetic community, native or spiked-in saliva and feces samples with qPCR**

In the simple synthetic community, the amplification of Caco2 and dead bacterial DNA by qPCR was decreased by PMAxx pretreatment in a dose-dependent manner. The amplification of live bacterial DNA was not affected (Table. S1). Whether the four spike-in bacteria existed in native saliva and feces samples were validated with qPCR. *E. coli, S. enterica*, *E. faecalis,* and *L. plantarum* were not found in three hosts’ saliva (Table. S2). For fecal samples, only *E. coli* was detected in three hosts and its quantity in host 1 was marginal (Table. S2). QPCR Ct values of the four bacteria in spiked-in saliva and feces samples were shown in Table S3 and S4. More than 95% of the spike-in dead microbes had been eliminated, and the living microbes were slightly affected.

1. **Preparation and confirmation of the high percentage of dead spike-in bacteria**

When exploring the heat-killed conditions, we measured the viable bacteria at different times of heat treatment. Logarithmic phase *E. coli* (7.32×10^7^CFU/ml ) was incubated at 96°C for 1min, 8min, and 15 min. And logarithmic phase *L. plantarum* (6.41×10^8^ CFU/ml) was incubated at 96°C for 15min and 20 min. The viable bacteria were measured with plate counting. No live *E. coli* and *L. plantarum* were detected via plate-counting after incubating at 96 ℃ for 15 min and 20 min. The results of these experiments are given in Table S6.

1. **Flow cytometry for the detection of the live/dead spike-in bacteria**

Except for plate counting, we validated the live/dead of the spike-in bacteria through SYTO 9 and propidium iodide staining and measuring with flow cytometry.

1 ml of the untreated and heat-killed bacterial suspensions were centrifuged at 10,000 × g for 1–3 min. And the pellets were resuspended in 1 ml of 0.85% NaCl. The suspensions were added with SYTO 9 (Thermo fisher, USA) and propidium iodide (Absin, Shanghai, China) dyes to the final concentration of 5 μm and 30 μm, and incubated at room temperature for 15 min protected from light as previously reported (1). The stained cells were detected on a Cytek Aurora flow cytometer (Cytek Biosciences, Fremont, CA), with 488 nm excitation from a blue solid-state laser at 50 mW. Green and red channel fluorescence were measured by the [fluorescence detector](javascript:;). Unstained and single-color (SYTO9/propidium iodide) stained bacterial cells were set as controls for setting up the flow cytometer.

Propidium iodide (PI) and SYTO9 staining showed the live/dead bacteria before and after heat treatment. PI^+^ cells, PI^+^SYTO9^+^ cells, and SYTO9^+^ cells were considered as dead, injured, and live cells, respectively (Fig. S4). The proportions of live bacteria were counted in table S7 at https://figshare.com/articles/dataset/The_proportions_of_live_bacteria_detected_with_flow_cytometry/21670967. Before heat treatment, more than 97.0% of the cells were alive. After heat treatment, more than 99.9% of the cells were dead.

1. **The death rates of the salivary and fecal bacterial species were associated with human health**

The salivary and fecal viable microbiome has a profound impact on human health. *Haemophilus parahaemolyticus* and *Aggregatibacter aphrophilus* were the most resistant species to lyPMAxx indicating highly alive/intact of these species in saliva (Fig. 5A). They were usually found in the human oropharynx and upper respiratory tract and were opportunistic pathogens leading to endocarditis (2-5). High ratio of live to dead of these microbes in saliva may indicate an increased risk of the disease. In feces samples of three hosts, *Gemella sanguinis* was the species with the largest fold changes by lyPMAxx, which was found on mucus membranes including the oropharynx, gastrointestinal tract, and genitourinary tract and associated with infections like endocarditis (6). Most of the other highly dead/injured microbes like *Eubacterium ramulus, Streptococcus salivarius, Roseburia hominis, Fusicatenibacter saccharivorans, Dorealongicatena, Roseburia inulinivorans, Roseburia intestinalis,* and *Coprococcus comes* belong to Lachnospiraceae. Lachnospiraceae contribute to the production of the short-chain fatty acids (SCFAs) (7, 8), resistance against drug-resistant pathogens through conversion of primary to secondary bile acids (9, 10), or production of peptide antibiotics lantibiotics (11, 12). A high ratio of dead/injured Lachnospiraceae was disadvantageous for human health. 5 species were reduced by less than 2.5 times and regarded as highly alive/intact bacteria in feces (Fig. 5C). Among them, *Bilophila wadsworthia* was reported to be associated with metabolic dysfunctions induced by high-fat diets (13). *Methanosphaera stadtmanae* and *Methanobrevibacter smithii* were major members of methanogen archaea, which contributed to the immune response in humans (14, 15).

1. **The association of the “Freezing -responsive” and “Freezing -resilient” species to human health**

*Actinobaculum sp. Oral taxon 183* was the most affected taxon by freezing in three host’ saliva. Among the most affected bacterial species by freezing in feces, *Bacteroides ovatus, Bacteroides intestinalis, Bacteroides thetaiotaomicron, Bacteroides xylanisolvens,* and *Bacteroides uniformis,* which belong to the genus of Bacteroides, have been reported to alleviate inflammation or help to regulate immunity (16, 17). *Eubacterium hallii, Roseburia faecis, Blautia wexlerae,* and *Anaerostipes hadrus* were Lachnospiraceae family, which contributed to the production of the SCFAs (7). Freeze-thaw reduced the absolute abundance of these bacteria by 25 times, which may reduce the efficiency of FMT. In addition, *Asaccharobacter celatus* was relatively resistant to freezing and its abundance decreased less than 2.5 times. It was reported to participate in isoflavone metabolism and be associated with equol production (18).

**Supplementary References**

1. Berney M, Hammes F, Bosshard F, Weilenmann HU, Egli T. 2007. Assessment and interpretation of bacterial viability by using the LIVE/DEAD BacLight Kit in combination with flow cytometry. Appl Environ Microbiol 73:3283-90.

2. González-Díaz A, Tubau F, Pinto M, Sierra Y, Cubero M, Càmara J, Ayats J, Bajanca-Lavado P, Ardanuy C, Marti S. 2019. Identification of polysaccharide capsules among extensively drug-resistant genitourinary Haemophilus parainfluenzae isolates. Scientific Reports 9:4481.

3. Le Floch AS, Cassir N, Hraiech S, Guervilly C, Papazian L, Rolain JM. 2013. Haemophilus parahaemolyticus septic shock after aspiration pneumonia, France. Emerg Infect Dis 19:1694-5.

4. Rempe KA, Spruce LA, Porsch EA, Seeholzer SH, Nørskov-Lauritsen N, St Geme JW, 3rd. 2015. Unconventional N-Linked Glycosylation Promotes Trimeric Autotransporter Function in Kingella kingae and Aggregatibacter aphrophilus. mBio 6.

5. Hidalgo-García L, Hurtado-Mingo A, Olbrich P, Moruno-Tirado A, Neth O, Obando I. 2015. Recurrent infective endocarditis due to Aggregatibacter aphrophilus and Staphylococcus lugdunensis. Klin Padiatr 227:89-92.

6. Emmanouilidou G, Voukelatou P, Vrettos I, Aftzi V, Dodos K, Koumpouli D, Avgeropoulos G, Kalliakmanis A. 2019. A Case Report of Successful Conservative Treatment for Infective Endocarditis Caused by <i>Gemella sanguinis</i>. Case Reports in Infectious Diseases 2019:9382395.

7. Rivera-Chávez F, Zhang LF, Faber F, Lopez CA, Byndloss MX, Olsan EE, Xu G, Velazquez EM, Lebrilla CB, Winter SE, Bäumler AJ. 2016. Depletion of Butyrate-Producing Clostridia from the Gut Microbiota Drives an Aerobic Luminal Expansion of Salmonella. Cell Host Microbe 19:443-54.

8. Byndloss MX, Olsan EE. 2017. Microbiota-activated PPAR-γ signaling inhibits dysbiotic Enterobacteriaceae expansion. 357:570-575.

9. Buffie CG, Bucci V, Stein RR, McKenney PT, Ling L, Gobourne A, No D, Liu H, Kinnebrew M, Viale A, Littmann E, van den Brink MRM, Jenq RR, Taur Y, Sander C, Cross JR, Toussaint NC, Xavier JB, Pamer EG. 2015. Precision microbiome reconstitution restores bile acid mediated resistance to Clostridium difficile. Nature 517:205-208.

10. Studer N, Desharnais L, Beutler M, Brugiroux S, Terrazos MA, Menin L, Schürch CM, McCoy KD, Kuehne SA, Minton NP, Stecher B, Bernier-Latmani R, Hapfelmeier S. 2016. Functional Intestinal Bile Acid 7α-Dehydroxylation by Clostridium scindens Associated with Protection from Clostridium difficile Infection in a Gnotobiotic Mouse Model. Front Cell Infect Microbiol 6:191.

11. Hatziioanou D, Gherghisan-Filip C, Saalbach G, Horn N, Wegmann U, Duncan SH, Flint HJ, Mayer MJ, Narbad A. 2017. Discovery of a novel lantibiotic nisin O from Blautia obeum A2-162, isolated from the human gastrointestinal tract. Microbiology (Reading) 163:1292-1305.

12. Kim SG, Becattini S, Moody TU, Shliaha PV, Littmann ER, Seok R, Gjonbalaj M, Eaton V, Fontana E, Amoretti L, Wright R, Caballero S, Wang ZX, Jung HJ, Morjaria SM, Leiner IM, Qin W, Ramos R, Cross JR, Narushima S, Honda K, Peled JU, Hendrickson RC, Taur Y, van den Brink MRM, Pamer EG. 2019. Microbiota-derived lantibiotic restores resistance against vancomycin-resistant Enterococcus. Nature 572:665-669.

13. Natividad JM, Lamas B, Pham HP, Michel M-L, Rainteau D, Bridonneau C, da Costa G, van Hylckama Vlieg J, Sovran B, Chamignon C, Planchais J, Richard ML, Langella P, Veiga P, Sokol H. 2018. Bilophila wadsworthia aggravates high fat diet induced metabolic dysfunctions in mice. Nature Communications 9:2802.

14. Camara A, Konate S, Tidjani Alou M, Kodio A, Togo AH, Cortaredona S, Henrissat B, Thera MA, Doumbo OK, Raoult D, Million M. 2021. Clinical evidence of the role of Methanobrevibacter smithii in severe acute malnutrition. Scientific Reports 11:5426.

15. Chaudhary PP, Conway PL, Schlundt J. 2018. Methanogens in humans: potentially beneficial or harmful for health. Appl Microbiol Biotechnol 102:3095-3104.

16. Ihekweazu FD, Engevik MA, Ruan W, Shi Z, Fultz R, Engevik KA, Chang-Graham AL, Freeborn J, Park ES, Venable S, Horvath TD, Haidacher SJ, Haag AM, Goodwin A, Schady DA, Hyser JM, Spinler JK, Liu Y, Versalovic J. 2021. Bacteroides ovatus Promotes IL-22 Production and Reduces Trinitrobenzene Sulfonic Acid-Driven Colonic Inflammation. Am J Pathol 191:704-719.

17. Li K, Hao Z, Du J, Gao Y, Yang S, Zhou Y. 2021. Bacteroides thetaiotaomicron relieves colon inflammation by activating aryl hydrocarbon receptor and modulating CD4(+)T cell homeostasis. Int Immunopharmacol 90:107183.

18. Iino C, Shimoyama T, Iino K, Yokoyama Y, Chinda D, Sakuraba H, Fukuda S, Nakaji S. 2019. Daidzein Intake Is Associated with Equol Producing Status through an Increase in the Intestinal Bacteria Responsible for Equol Production. Nutrients 11:433.
